# Naked metallic skin for homo-epitaxial deposition in lithium metal batteries

**DOI:** 10.1038/s41467-023-36934-x

**Published:** 2023-03-09

**Authors:** Minsung Baek, Jinyoung Kim, Kwanghoon Jeong, Seonmo Yang, Heejin Kim, Jimin Lee, Minkwan Kim, Ki Jae Kim, Jang Wook Choi

**Affiliations:** 1 School of Chemical and Biological Engineering and Institute of Chemical Processes, Seoul National University, Seoul, Republic of Korea; 2 Department of Chemistry, Seoul National University, Seoul, Republic of Korea; 3 Electron Microscopy Research Center, Korea Basic Science Institute, Daejeon, Republic of Korea; 4 Department of Energy Engineering, Konkuk University, Seoul, Republic of Korea; 5 Department of Materials Science and Engineering, Seoul National University, Seoul, Republic of Korea

**Keywords:** Batteries, Batteries, Batteries

## Abstract

Regulating the morphology of lithium plating is the key to extending the cycle life of lithium metal batteries. Fatal dendritic growth is closely related to out-of-plane nucleation on the lithium metal surface. Herein, we report a nearly perfect lattice match between the lithium metal foil and lithium deposits by removing the native oxide layer using simple bromine-based acid-base chemistry. The naked lithium surface induces homo-epitaxial lithium plating with columnar morphologies and lower overpotentials. Using the naked lithium foil, the lithium-lithium symmetric cell maintains stable cycling at 10 mA cm^−2^ for more than 10,000 cycles, and the full-cell paired with LiFePO_4_ with high areal capacity of 3.3 mAh cm^−2^ and practical N/P ratio of 2.5 exhibits 86% capacity retention after 300 cycles. This study elucidates the usefulness of controlling the initial surface state to facilitate homo-epitaxial lithium plating for sustainable cycling of lithium metal batteries.

## Abstract (web summary)

The oxide passivation layer on the lithium metal surface causes uneven deposition and stripping in lithium metal batteries. Here authors introduce uniform homo-epitaxial lithium deposition on the metal surface to alleviate this issue and improve the cycle stability of the lithium metal batteries.

## Introduction

The surging availability of electric vehicles (EVs) and grid-scale utility storage coupled to renewable energy conversion systems has considerably increased the demand for rechargeable batteries with high energy density^1–4^. Given that the practical specific capacity of the graphite anode in existing lithium-ion batteries (LIBs) has saturated near its theoretical limit (372 mAh g^−1^), an intensive search is underway to identify anode materials with unparalleled specific capacities^5–7^. Among several promising candidates, metallic lithium (Li) stands out based on its low operating voltage (−3.04 V vs. the standard hydrogen electrode) and extraordinarily high specific capacity (3860 mAh g^−1^)^8,9^. However, rechargeable Li metal batteries (LMBs) are hardly adopted in practical cells due to the inherent physicochemical properties of Li metal. That is, the Li metal surface is susceptible to dendritic growth, which is accompanied with the indiscriminate chemical, electrochemical decomposition of the electrolyte. These phenomena ruin the lifetime and safety of a battery cell severely, and detailed mechanisms and analyses were recently disclosed in numerous reports^10–14^.

The mechanism behind the dendritic growth of Li metal involves various parameters related to the bath conditions and substrate. The bath conditions are represented^15–18^ by the pressure, temperature, and electrolyte inside the cell and have a direct influence on the morphology of Li electro-deposits. In particular, electrolyte engineering has turned out to be effective in flattening the Li electro-deposits. Nonetheless, the crystallographic characteristics of Li electro-deposits may not be controlled solely by the electrolyte composition but rather comprehensively based on the interfacial energy minimization between the electro-deposited metal and the substrate of choice. This rationale explains why in-plane nucleation is scarcely realized in LMBs in which the Li metal anode forms an incoherent interface with high interfacial energy. Instead, the incoherent interface induces the out-of-plane nucleation, as in most of the lattice-mismatching systems^19^.

In this regard, substrate engineering^20–22^ appears to be a feasible approach to control the nucleation of Li deposits and thus regulate their growth direction. In particular, the interfacial energy between a newly deposited layer and a substrate with similar lattice parameters would induce minimal strain and thus impose the least interfacial energy, increasing the possibility of in-plane nucleation. Furthermore, with negligible lattice misfit, the crystallographic orientation of the deposit could be aligned to that of the substrate, a phenomenon known as epitaxy. Along this direction, Archer et al. demonstrated that the alignment of graphene by shear force can serve as a basis on which to develop the so-called hetero-epitaxial growth of zinc metal; that is, the epilayer and the substrate are composed of different (hetero-) materials^23^. The epitaxy can be divided further into single crystalline epitaxy and polycrystalline epitaxy depending on the crystallinity of the substrate.

With LMBs, homo-epitaxy (Li^0^ on Li^0^) is desired over hetero-epitaxy (Li^0^ on others) because homo-epitaxial templating does not require foreign templates to induce homogeneous deposition. In this way, the high energy density of LMBs is not sacrificed. Although the Li^0^ substrate is suitable for promoting the polycrystalline-rooted epitaxial deposition of incoming Li ions, which would allow a lattice match, homo-epitaxial deposition of this nature has hardly been realized owing to the existence of the native Li_2_O layer on the Li foil surface. The presence of the native Li_2_O layer is attributed to the reactive nature of metallic Li. The Li_2_O layer not only bears significantly lower ionic conductivity compared to the electrochemically driven SEI layer but also constitutes inhomogeneity in passivation, resulting in the preference for irregular sites with regard to the nucleation of Li metal^24^. Accordingly, electro-deposition occurs favorably at fragile spots with lean Li_2_O, allowing the Li metal to develop a coherent interface only in limited regions. The resulting nucleation in small local areas causes the surface charges to localize and accelerate dendritic growth^25,26^. All in all, in order to fully utilize the intrinsic crystal structure of the substrate and thus ensure that the homo-epitaxial deposits are densely packed, the nucleation environments need to be carefully taken into consideration.

**Fig. 1:**
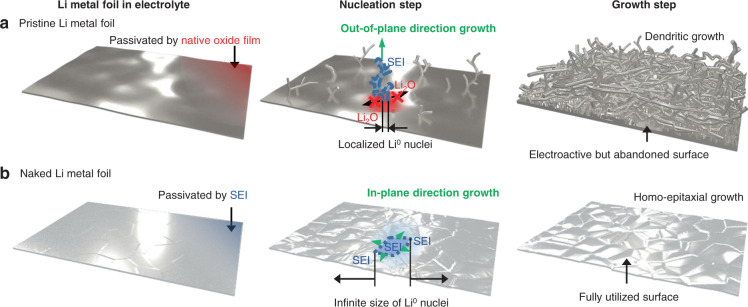
Importance of the surface state in controlling the morphology of Li deposit. Schematic illustration of the nucleation and growth steps of **a**, the pristine and **b**, naked Li metal foils. The red and blue shading represents the existence of the native oxide film and electrolyte-based SEI, respectively. The green arrows signify the dominant direction for electrodeposition: out-of-plane and in-plane directional growth for the pristine and naked Li foils, respectively.

Figure 1 schematically illustrates the importance of the surface condition in developing facile nucleation with uniform coverage. In general, the lithium oxide components (Li_2_O, Li_2_O_2_) natively formed on the Li foil are known to impose substantially high resistance for Li ion diffusion compared to the solid-electrolyte-interphase (SEI) layer produced by the (electro)chemical reaction with the electrolyte. This high ionic resistance of the lithium oxide layer causes the Li deposition to be distributed more unevenly (Fig. 1a) as cycling continues; once the initial deposits are formed by penetrating the lithium oxide layer, the subsequently incoming Li ions prefer to nucleate on the existing deposits (covered with the SEI) rather than newly penetrating the lithium oxide layer. Consequently, the electroactive but initially unutilized foil surfaces remain abandoned while the out-of-plane growth on top of the native oxide layer is accelerated to form mossy layers of deposit. That being said, we hypothesized that stripping the native oxide layer and replacing it with the electrolyte-driven SEI layer would prevent the nucleation distribution from becoming uneven (Fig. 1b). This was expected to confine the growth of nuclei to the in-plane direction such that the crystalline orientations of the Li deposits are aligned to the original Li substrate. As a result, densely packed homo-epitaxy would serve to guide the deposition throughout cycling and therefore improve the cyclability markedly.

We realized that certain surface-sensitive systems such as semiconductor devices have adopted an etching step to remove the native surface layer before applying a coating of any material to warrant the quality of the coating layer^27^. However, in LMBs, the effect of the native passivation layer has largely been ignored because eliminating this layer without it being re-oxidized is experimentally nontrivial. Researchers employed chemical polishing^28^, mechanical destruction^29^, and electrochemical stripping^30^ to remove the Li_2_O layer, but maintaining the naked metallic Li skin throughout cell fabrication and cycling was still unattainable.

In this study, we introduce a chemical etchant based on a protection-deprotection approach to implement the naked Li foil in a cell without re-oxidation. To this end, the Li_2_O passivation layer was stripped and replaced by a lithium bromide (LiBr) layer via an elaborate acid-base reaction. The LiBr layer was then washed away by the electrolyte solution immediately before the passivation-free Li metal foil was integrated into a cell without exposure to oxygen. This simple yet effective treatment enables epitaxial plating of Li metal directly on the bare Li metal skin, delivering exceptionally stable cycling over 10,000 cycles even at an extraordinarily high current density of 10 mA cm^−2^.

## Results

### Realizing homo-epitaxy through naked metallic skin

**Fig. 2:**
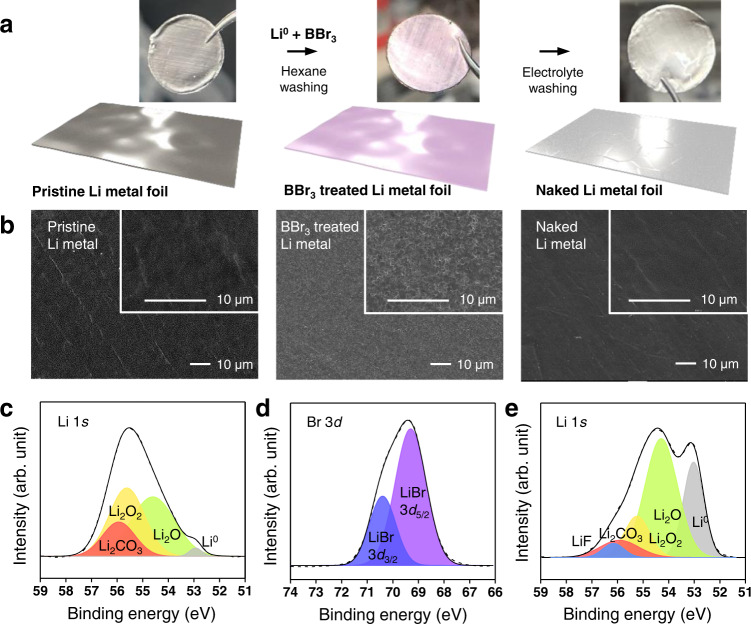
Characterization of the pristine, BBr_3_-treated, and naked Li metal foils. **a** Schematic illustration of the elimination of the native film with photographic images of the Li metal foils after individual reaction steps: in its pristine state (left), after treatment with BBr_3_ (middle), after washing with the electrolyte of choice (right). **b** SEM images of Li metal foils after the corresponding reaction steps (the insets are the magnifications). **c** Li 1 *s* XPS plot of the pristine Li metal foil. **d** Br 3*d* XPS plot of the BBr_3_-treated Li metal foil. **e** Li 1 *s* XPS plot of the naked Li metal foil.

To take full advantage of the coherent interface between the substrate and the deposition layer, the intrinsic lithium oxide layer on the Li foil needs to be removed. However, because naked Li is very reactive even with traces of oxygen in a well-maintained glove box, we covered the native Li metal skin with a layer of LiBr to prevent re-oxidation of the Li metal. LiBr was carefully chosen because it is chemically stable in contact with metallic Li and can also be readily removed with the commonly used carbonate or ether electrolytes. Specifically, boron tribromide (BBr_3_), a liquid bromination reagent, was reacted with the Li metal foil via an acid-base reaction to remove the lithium oxide layer from the surface. The resultant LiBr layer was subsequently dissolved by immersing the Li foil in the electrolyte (Fig. 2a) to expose the metallic Li skin. Notably, BBr_3_ is a very strong Lewis acid owing to its D_3h_ symmetry of which the z-axis orbitals are vacant. Because of its strong electron-withdrawing ability, BBr_3_ not only reacts violently with Lewis basic Li^0^ (Supplementary Fig. 1) but also partially with lithium oxide (Supplementary Fig. 2). In this reaction, the BBr_3_ reagent permeates through grain boundaries and cracks in the native lithium oxide layer to react with the metallic Li and form a LiBr layer directly on top of Li^0^, exerting mechanical stress to disintegrate the passivation layer:

1 $$2{{{{{{\rm{Li}}}}}}}^{0}+2{{{{{{\rm{BBr}}}}}}}_{3}\to {{{{{{\rm{B}}}}}}}_{2}{{{{{{\rm{Br}}}}}}}_{4}+2{{{{{\rm{LiBr}}}}}}$$

Because the reaction is highly vigorous, the reactivity was controlled by dissolving BBr_3_ in a nonpolar solvent, namely hexane, while the entire process was undertaken inside a glove box under argon atmosphere. Hexane, which is composed solely of aliphatic chains, was able to keep the native skin of Li metal intact without undergoing any side reactions. In addition, the targeted LiBr layer is insoluble in hexane and is therefore able to protect the metallic skin against oxidation as well as the aggressive reaction with BBr_3_. As soon as the LiBr layer was in place, the gray tint of the pristine Li foil became light pink (Fig. 2a, middle). As the LiBr coating was washed away in the electrolyte, 1 M lithium hexafluorophosphate (LiPF_6_) in ethylene carbonate/diethyl carbonate (EC/DEC = 50/50 (v/v)) with 10 wt% fluoroethylene carbonate (FEC) (1 M LiPF_6_ EC/DEC 10 wt% FEC), immediately before cell assembly, the exposed Li metal skin was minimally exposed to impurities in the glove box. During this washing and transfer process, the Li metal skin was anticipated to form an electrolyte-derived SEI layer that would determine the nucleation behavior along the metallic skin underneath. We use the term ‘naked’ even for the Li metal surface covered by an SEI layer because this surface state is distinct from typical Li metal surface passivated by oxide layer.

Field emission scanning electron microscopy (FE-SEM) was used (Fig. 2b) to visualize the Li metal surface at different stages of the surface treatment. Notably, the BBr_3_-treated sample was covered with a uniform layer of a certain morphology, which reflected that of the LiBr crystal. After washing with the electrolyte, the LiBr surface morphology disappeared to reveal the clean, featureless Li metallic skin. The chemical components of the electrode surfaces at each stage were investigated by analyzing the electrodes with X-ray photoelectron spectroscopy (XPS). In the case of the pristine Li foil, consistent with the literature^31^, the native surface layer was composed of Li_2_CO_3_, Li_2_O_2_, and Li_2_O, which were detected at 56.0, 55.6, and 54.6 eV in the Li 1 s branch, respectively (Fig. 2c). After the treatment with BBr_3_, peaks assigned to LiBr and LiB_x_O_y_ were clearly observed at 69.3 and 70.4 eV in the Br 3*d* branch and 192.4 eV in the B 1 s branch (Fig. 2d and Supplementary Fig. 3b), confirming the aforementioned reaction scheme. After the washing step with the electrolyte, the peaks corresponding to LiBr and LiB_*x*_O_*y*_ were no longer observed in the Br 3*d* and B 1 *s* bands, validating the high solubility of the targeted LiBr layer in the electrolyte solution. Noticeable is the significant increase in the ratio of the metallic Li peak at 53.0 eV in the Li 1 *s* band to 24.9% from 1.2% for the naked and pristine state, respectively (Fig. 2e). Even after the 1^st^ electrodeposition on the naked Li metal foil, the metallic Li peak was still largely maintained (Supplementary Fig. 4), implying that the SEI layer formed is very thin. It should be noted that although sample preparation and delivery for XPS analysis was carried out in an argon atmosphere, the metallic surface was inevitably exposed to impurities therein, which led us to anticipate that the portion of the metallic Li actually transferred to the cells would be much higher. Overall, passivation with the chemically driven SEI layer is the key to keeping the metallic Li skin unburied during cycling.

**Fig. 3:**
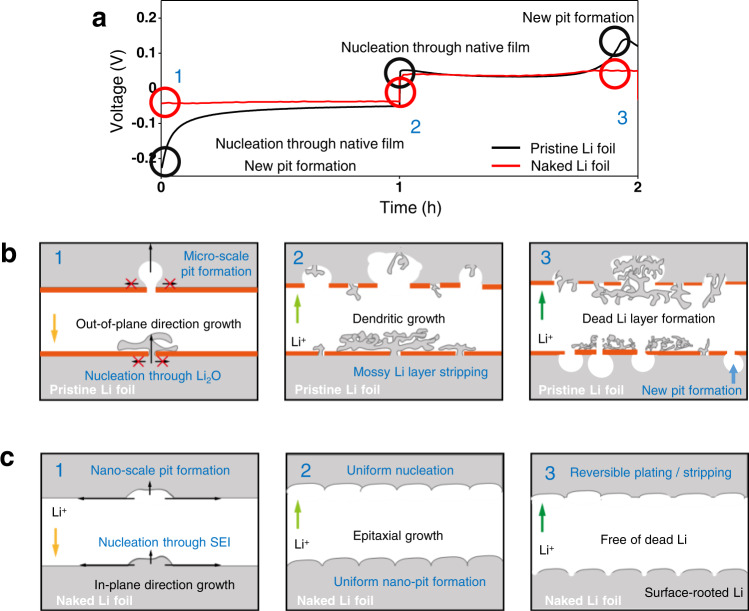
Correlation between the surface state of Li foil and the potential profile in the first cycle. **a** Galvanostatic charge-discharge voltage profiles of the pristine (black) and naked (red) Li-Li symmetric cells in the first cycle. Schematic illustrations of electrode morphology at different points in the first galvanostatic charge-discharge cycle: **b** the pristine Li foil and **c** the naked Li foil. The current density and areal capacity are 1 mA cm^−2^ and 1 mAh cm^−2^, respectively.

Indeed, the first charging and discharging profiles reflect the surface characteristics of the Li metal electrodes effectively (Fig. 3a). For these tests, Li-Li symmetric cells were fabricated using the electrolyte 1 M LiPF_6_ EC/DEC (50/50 = v/v) with 10 wt% FEC and were galvanostatically scanned at a constant current density of 1 mA cm^−2^ with 1 mAh cm^−2^. Stages 1, 2, and 3 (Fig. 3b, c) can be correlated with the corresponding points in the galvanostatic profiles: In stage 1, the nucleation overpotential of the pristine Li cell was much larger than that of its naked Li cell counterpart. This phenomenon can be explained by the effect of the native film that imposes additional resistance related to the penetration of Li ions at both the working and counter electrodes^32^. This resistance related to the penetration of Li ions is absent from the naked Li cell. As stage 1 gradually fades away, the overpotential of the pristine Li cell approaches that of the naked Li cell as the Li ions deposit on the dendrites covered by SEI. When the current is reversed in stage 2, the pre-deposited dendrites, instead of the metallic Li underneath the native oxide layer, are preferentially stripped at the working electrode, while nucleation at the counter electrode occurs at the previously formed micro-pits, resulting in a much lower overpotential compared to stage 1. Nevertheless, as indicated by the average Coulombic efficiency of <100%, the pre-deposited Li layer at the working electrode becomes depleted before the given measurement step ends, and nucleation through the native film is re-initiated (new pit formation) as in stage 1, resulting in the voltage spike in stage 3. Owing to the absence of the native oxide layer, the overpotential of the naked Li cell did not increase during these three stages. The surface of the naked Li metal was covered solely by the SEI layer through which Li ions diffuse. As is clear from the cyclic voltammetry (CV) results acquired in the limited range of −200 mV to +200 mV (Supplementary Fig. 5), the symmetric cell with the pristine Li foil hardly exhibited a current flow, verifying the necessity of the large overpotential to enable the Li ions to penetrate the native oxide layer for initial electrodeposition. By contrast, the current began to flow in the naked Li cell immediately above and below −20 mV and +20 mV, respectively.

**Fig. 4:**
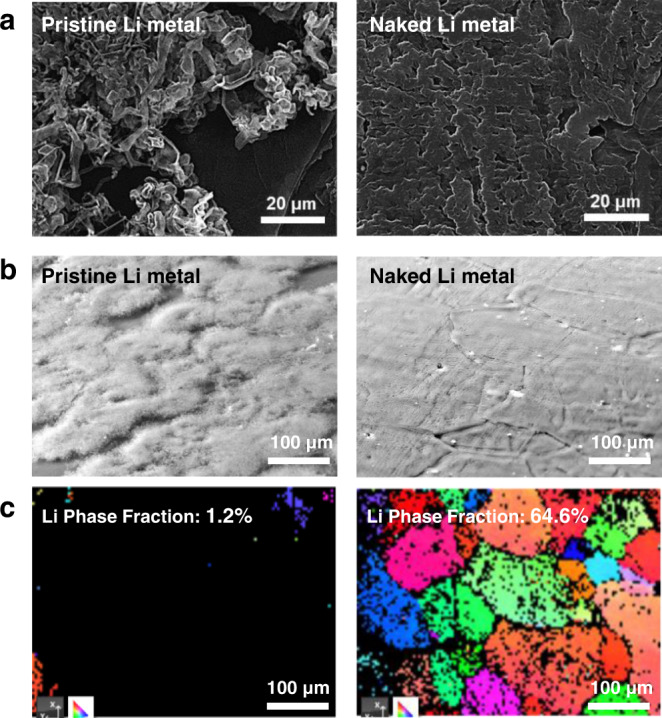
Effect of naked Li surface on the morphology of the Li deposit. **a** SEM images of the pristine (left) and naked (right) Li metal foils after galvanostatic electrodeposition at 1 mA cm^−2^ with 1 mAh cm^−2^. **b** Slanted SEM images and the corresponding **c** EBSD images of the pristine (left) and naked (right) Li metal foils after galvanostatic electrodeposition at 1 mA cm^−2^ with 1 mAh cm^−2^.

Removal of the native film not only lowers the nucleation energy but also induces uniform Li deposition by unifying the initial passivation with SEI as explained in Fig. 1. Once epitaxial Li deposition becomes infeasible owing to the presence of the surface passivation layer, the Li deposits dominantly have a whisker-like morphology, which accelerates dendritic growth. Thus, the availability of the naked Li surface is pivotal to determining the morphology of Li deposits and therefore charge-discharge reversibility. To verify this perception, both the pristine and BBr_3_-treated Li foils were imaged employing FE-SEM analysis after galvanostatically plating 1 mAh cm^−2^ Li at a current density of 1 mA cm^−2^ (Fig. 4). As discussed above, the presence of the native oxide layer causes Li deposits to irregularly nucleate over the electrode area, resulting in areas distinctly plated and unplated with Li (Fig. 4a, left). By contrast, the plated Li on the naked Li metal foil covered the entire substrate (Fig. 4a, right), reflecting the uniform and regular nucleation of the Li deposit. Because the contrast between the areas plated and unplated with Li becomes more pronounced when observed at a tilted angle, both samples were imaged at 70° at lower magnification (Fig. 4b). This visualization further confirmed the uniform coverage of the Li deposit in the case of the naked Li foil sample. Remarkably, this trend was preserved even at the high areal capacities of 5 and 10 mAh cm^−2^ (Supplementary Fig. 6) at which the densely packed morphology of the Li deposits was observed during operation. When imaged at high magnifications (Supplementary Fig. 7), the significant growth of the SEI layer along the Li dendrites on the pristine Li foil was captured by brightness owing to the charging effect, unlike the naked Li metal electrode. The distinct morphology with respect to the uniformity of the surface structure was also observed after Li stripping (Supplementary Fig. 8), after which the naked Li metal electrode exhibited clean featureless morphology whereas the surface of the pristine Li metal electrode displayed a number of deep micro-pits.

To further investigate the orientations of the Li deposits, electron back-scatter diffraction (EBSD) analysis was conducted to capture the diffraction patterns of the metallic surface and quantify the extent to which the crystal orientation of the original metal surface is preserved after Li metal plating. The EBSD analysis (Kikuchi line) detects only the top surface to a depth of about 50 nm^33^, thus a diffraction pattern can be obtained only when the passivation layer of the Li deposit layer is sufficiently thin. After 1 mAh cm^−2^ Li was plated at 1 mA cm^−2^, only 1.2% of the area of the pristine Li metal electrode matched that of the original Li metal surface whereas the value was much higher for the naked Li metal electrode (64.6%) (Fig. 4c). The orientation mapping of the naked Li metal electrode indicates that the electrodeposition layer grew homo-epitaxially to preserve the crystal orientation of the grains in the original Li metal foil. The different colors refer to polycrystalline grains with the same crystal structures but different crystal orientations, pointing to the polycrystalline homo-epitaxial electrodeposition of Li deposit. On the other hand, the negligible trace signal of the pristine Li metal electrode is attributed to the electrodeposition layer with random crystal orientations.

### Electrochemical performance

**Fig. 5:**
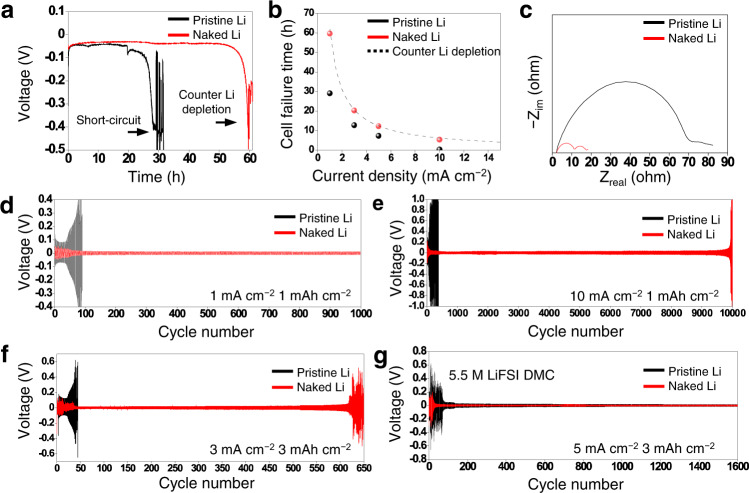
Electrochemical test results of symmetric cells. **a** Chronopotentiometry measurements of the pristine (black) and naked (red) Li-Li symmetric cells at a current density of 1 mA cm^−2^. **b** Cell failure time for the pristine (black) and naked (red) Li-Li symmetric cells during chronopotentiometry measurements at various current densities. The dashed line indicates the theoretical depletion time of the Li metal counter electrode with the thickness of 300 μm at each current density. **c** Nyquist plots of the pristine (black) and naked (red) Li-Li symmetric cells before galvanostatic cycling. Galvanostatic charge-discharge voltage profiles of the pristine (black) and naked (red) Li-Li symmetric cells with operating conditions of **d** 1 mA cm^−2^, 1 mAh cm^−2^, **e** 10 mA cm^−2^, 1 mAh cm^−2^, **f** 3 mA cm^−2^, 3 mAh cm^−2^, and **g** 5 mA cm^−2^, 3 mAh cm^−2^. Electrolytes: 1 M LiPF_6_ in EC/DEC (50/50 = v/v) with 10 wt % FEC for **d**−**f**, and 5.5 M LiFSI in DMC for **g**.

The electrochemical performance of these two Li metal foils was validated by fabricating Li-Li symmetric cells using the electrolyte of 1 M LiPF_6_ EC/DEC (50/50 = v/v) with 10 wt% FEC. The homo-epitaxial electrodeposition of the naked Li foil was expected to largely inhibit the formation of dendrites and thus internal short-circuits. To validate this scenario by particularly focusing on the occurrence of short-circuits, a chronopotentiometry test was performed at various current densities from 1 mA cm^−2^ to 10 mA cm^−2^. When tested at 1 mA cm^−2^ (Fig. 5a), the symmetric cell with the naked Li foil maintained a stable voltage profile for >61 h until the counter electrode was depleted. For reference, the theoretical depletion time based on the foil thickness is 61.9 h. By contrast, the pristine Li foil ceased to operate after only 29 h owing to short-circuiting. The superior stability of the naked Li foil was preserved at the higher current densities with lower electrodeposition overpotentials (Supplementary Fig. 9). The failure time is collectively plotted against the different current densities (Fig. 5b) where the theoretical cell failure time associated with Li metal depletion is drawn together in the dashed line considering the thickness of the Li foil (300 μm) that corresponds to 61.9 mAh cm^−2^. As displayed in Fig. 5b, the failure times of the naked Li cell coincided with the dashed line, implying that the lifetime of this cell was determined mainly by the Li depletion on the counter electrode. Unlike the naked Li cell, the pristine Li cell failed owing to short-circuiting initiated by the dendrite growth on the working electrode.

Electrochemical impedance spectroscopy (EIS) was used to analyze the Li-Li symmetric cells to confirm the effect of removing the native passivation layer on the ionic resistance. Before cycling, the impedance of the naked Li symmetric cell showed that the interfacial resistance was four times lower compared to that of its pristine Li counterpart (Fig. 5c). The first semi-circle (on the left) corresponds to the passivation layer resistance at the interface between the foil and the passivation layer and the second semi-circle (on the right) corresponds to the charge transfer resistance. Notably, the first semi-circle of the naked Li cell is significantly smaller than that of the pristine Li cell. This can be explained by the fact that the SEI layer on the naked Li foil, formed upon immersion in the electrolyte, is beneficial for facile Li ion transfer compared to the passivation layer (Li_2_O) of the pristine Li foil.

Next, the reversibility of Li plating and stripping was tested at various current densities (1−10 mA cm^−2^) for a range of areal capacities (1−3 mAh cm^−2^) (Fig. 5d–g). In all the areal current density and capacity ranges, the naked Li symmetric cell exhibited far more robust cycling performance than the pristine Li symmetric cell. For example, at 1 mA cm^−2^ with 1 mAh cm^−2^ (Fig. 5d), the overpotential of the cell with the pristine Li foil was high (~0.2 V) for the initial nucleation and became highly divergent upon cycling; the overpotential abruptly began to increase near the 58^th^ cycle and the cell ceased to operate before the 100^th^ cycle due to short-circuit formation, which was also reflected in the deformed semi-circle in the low frequency regime of its EIS profile (Supplementary Fig. 10 and Supplementary Table 1). In contrast, the initial nucleation overpotential of the naked Li cell was only 30 mV and the overpotential remained fairly stable for >1000 cycles. In fact, the overpotential of the naked Li cell decreased during the first 100 cycles owing to the increased electroactive surface area, which is discussed in detail later. The superior cycling performance of the naked Li symmetric cell based on the facile nucleation and subsequent homo-epitaxial plating was mirrored by the other areal current density and capacity conditions including 10 mA cm^−2^ and 1 mAh cm^−2^ (Fig. 5e), 3 mA cm^−2^ and 3 mAh cm^−2^ (Fig. 5f), and 5 mA cm^−2^ and 3 mAh cm^−2^ (Supplementary Fig. 11). The conditions in Fig. 5f (3 mA cm^−2^ and 3 mAh cm^−2^) benchmark the operating protocol for commercial cells based on graphite anodes. The cells yielding the results in Fig. 5g took advantage of the high concentration effect^34–38^ by employing 5.5 M lithium bis(fluorosulfonyl)imide (LiFSI) salt to overcome the tough operating conditions (5 mA cm^−2^ and 3 mAh cm^−2^). Both cells needed a stabilization period of ~50 cycles because of the high electrolyte concentration. Nevertheless, the initial overpotentials of the pristine and naked Li metal electrodes were distinct at 0.62 and 0.22 V, respectively, at this high current density. Recall that increasing the charging rate is nontrivial for LMBs because the Li ion flux near the Li metal foil readily becomes non-uniform; the higher current density shortens the so-called sand’s time at which Li ions are completely depleted^39–43^. Ultimately, the non-uniform Li ion flux leads to dendritic growth and cell failure. In this sense, removal of the surface passivation layer to ensure robust cycling performance at the current density of 10 mA cm^−2^ is fairly notable even compared to other studies that engaged 3D heteroatom hosts (Supplementary Table 2).

**Fig. 6:**
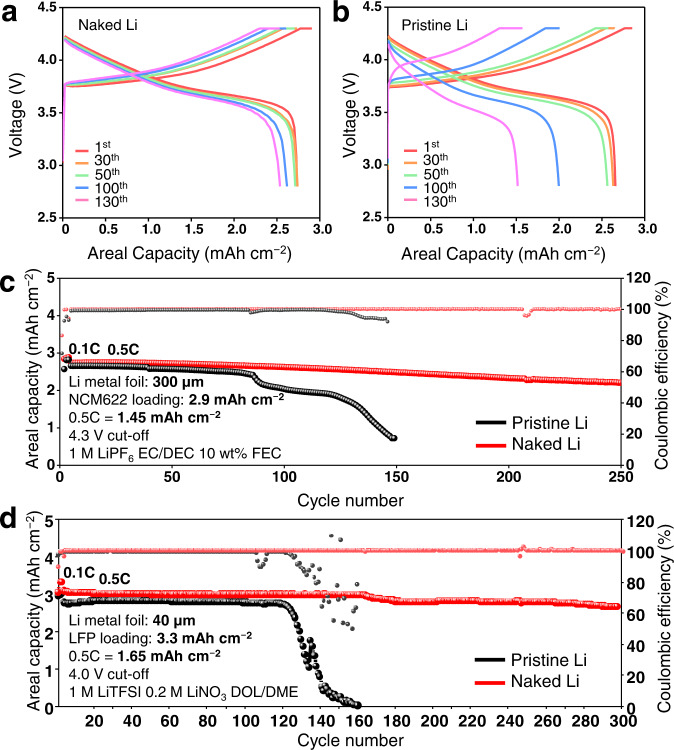
Electrochemical test results of full cells. Galvanostatic potential profiles of the full cells fabricated with 2.9 mAh cm^−2^ NCM622 as cathode and 300 µm thick **a**, naked and **b**, pristine Li metal foils as anodes for a different number of cycles. **c** Discharge capacity and Coulombic efficiency against the cycle number of full cells with 2.9 mAh cm^−2^ NCM622 as the cathode and 300 µm pristine (black) and naked (red) Li metal foils as anodes. 1 M LiPF_6_ in EC/DEC (50/50 = v/v) with 10 wt % FEC was used as electrolyte and the cells were cycled at 0.5 C (1.45 mA cm^−2^) after 3 pre-cycles at 0.1 C (0.29 mA cm^−2^) in the potential range of 2.8 − 4.3 V. **d** Discharge capacity and Coulombic efficiency against the cycle number of full cells with 3.3 mAh cm^−2^ LFP as the cathode and 40 µm pristine (black) and naked (red) Li metal foils as anodes. 1 M LiTFSI + 0.2 M LiNO_3_ in DOL/DME (50/50 = v/v) was used as electrolyte and the cells were cycled at 0.5 C (1.65 mA cm^−2^) after 3 pre-cycles at 0.1 C (0.33 mA cm^−2^) in the potential range of 2.5 − 4.0 V.

The excellent reversibility of the naked Li metal electrode (300 μm thick) prompted us to evaluate full cells by pairing this electrode with a lithium nickel manganese cobalt oxide (LiNi_0.6_Co_0.2_Mn_0.2_O_2_ or NCM622) cathode (active material: super P: binder = 95: 3: 2 by weight). To test the practical viability, the areal capacity of the full cells was set to 2.9 mAh cm^−2^ at the 0.1C-rate by loading 17.3 mg cm^−2^ of the cathode material. When cycled at 0.5 C (1.45 mA cm^−2^), the ability of the naked Li metal full cell to preserve the original potential profiles (Fig. 6a) was superior to that of its pristine Li metal counterpart (Fig. 6b). The more prominent capacity fading of the pristine Li metal full cell is ascribed to the higher overpotential resulting from SEI growth in conjunction with the formation of dead Li. This distinct sustainability of the Li metal anodes made a substantial difference in the cycle life such that when cycled at 0.5 C, the naked Li metal full cell retained 80.6% of its original capacity after 250 cycles whereas the pristine Li metal full cell lost its capacity quite abruptly after 86 cycles and preserved less than half of the initial capacity after 140 cycles (Fig. 6c).

The electrochemical evaluation was extended to more challenging conditions in which the amount of Li was restricted; the N/P ratio was set to 2.8 by employing Li foil with a thickness of 40 μm and the areal loading of NCM622 (17.3 mg cm^−2^, 2.9 mAh cm^−2^). Notably, the specific capacity of the Li metal anode with these electrode conditions corresponds to 1378 mAh g_Li_^−1^. Under these conditions, the capacity of the pristine Li full cell decayed rapidly around the 40^th^ cycle, whereas the naked Li full cell maintained its capacity and Coulombic efficiency much more stably for 180 cycles (Supplementary Fig. 12). As carbonate-based electrolytes are well known to be less stable with Li metal anodes, an ether-based electrolyte (1 M lithium bis(trifluoromethanesulfonyl)imide (LiTFSI)) + 0.2 M LiNO_3_ in 1,3-dioxolane (DOL)/ 1,2-dimethoxyethane (DME) (50/50 = v/v) was used for testing full cells paired with LiFePO_4_ (LFP) cathodes (Supplementary Fig. 13). For these tests, the N/P ratio was further reduced to 2.5 by pairing the 40 μm Li metal foil with high loading LFP cathode (21.9 mg cm^−2^, 3.3 mAh cm^−2^). The naked Li full cell retained >86% after 300 cycles at a high current density of 1.65 mA cm^−2^ while the pristine Li full cell decayed only after 100 cycles under the same conditions (Fig. 6d). Remarkably, this result is one of the best cycling performances reported to date in LMBs with practically viable N/P ratios (Supplementary Fig. 14 and Supplementary Table 3). When EIS analysis was performed before cycling and after 50, 100, and 200 cycles, the anode charge-transfer resistance increased sharply after 200 cycles for the pristine Li cell due to severe accumulation of dead Li, while such phenomenon was much less severe for the naked Li cell (Supplementary Fig. 15 and Supplementary Table 4). The cathode charge-transfer resistances of both cells hardly changed, suggesting that the cell failure of the pristine cell was mainly caused by dead Li accumulation in the anode.

The present approach of removing the native passivation layer differs from the widely used approaches in the literature that rely on the introduction of a protection layer on top of the pristine Li foil. Although our approach contrasts existing approaches with respect to the way in which the original Li foil is manipulated, the performance demonstrated in this study largely validates the effect of eliminating the passivation layer in resolving the chronic problems of Li metal electrodes. Moreover, our approach can be combined with other available strategies such as the incorporation of Li hosts^44–46^, solid electrolytes^47,48^, and an artificial SEI^49–51^ to further improve the cyclability.

### Influence on the deposition layer

**Fig. 7:**
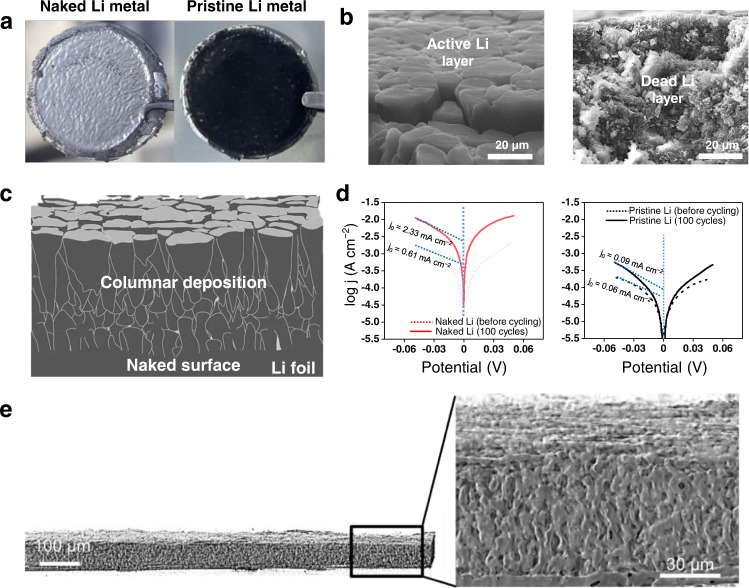
Influence of naked Li surface on the deposition layer. **a** Photographs of the naked (left) and pristine (right) Li metal foils after 100^th^ Li plating. Cycling conditions: 1 mA cm^−2^, 1 mAh cm^−2^. **b** SEM images of the active Li layer (left) on the previously naked Li metal foil and the dead Li layer (right) on the previously pristine Li metal foil after 100 cycles at 1 mA cm^−2^ with 1 mAh cm^−2^. **c** Schematic illustration of layer of columnar Li deposit grown on the naked Li foil. **d** Exchange current density of the naked (red) and pristine (black) Li-Li symmetric cells before (dashed line) and after (solid line) 100 cycles at 1 mA cm^−2^ with 1 mAh cm^−2^. **e** Reconstructed XTM images of the naked Li metal foil after 100 cycles at 1 mA cm^−2^ with 1 mAh cm^−2^.

The substantial improvement of the naked Li metal cells with regard to their cycle life stresses the importance of controlling the Li plating in the very beginning. The initial Li plating and stripping behavior affects the reversibility of the subsequent cycles and ultimately the cycle life of the corresponding cell. To further elucidate this rationale, post-mortem analyses were conducted by disassembling the pristine and naked Li cells in the carbonate-based electrolyte after 100 cycles at 1 mA cm^−2^ and 1 mAh cm^−2^. FE-SEM images acquired after the 100^th^ Li stripping indicated that the thickness of the Li deposition layer of the naked Li foil was more than two-fold smaller (Supplementary Fig. 16), 64.0 μm vs. 138.4 μm, than that of the pristine Li foil. The stability difference during Li plating-stripping cycling was reflected in the color of the Li foils (Fig. 7a). After the 100^th^ Li plating, a glossy silver appearance indicative of metallic Li was scattered over the entire area of the naked Li foil whereas the pristine Li foil was blackened. When imaged at a tilted angle, the Li deposits of the two Li foils displayed distinct morphologies (Fig. 7b and Supplementary Fig. 17). The Li deposits on the naked Li foil consisted of large columnar chunks originating from the initial epitaxial growth of Li metal as schematically illustrated (Fig. 7c). By contrast, the morphology of the Li deposits on the pristine Li foil was erratic (Fig. 7b right), and was covered by an indiscriminately grown SEI layer. When XPS analysis was conducted after the 100^th^ Li plating, the naked Li metal foil exhibited a relatively large portion of LiF-containing inorganic components in the SEI, while the pristine Li metal foil exhibited solvent-driven organic-rich composition (Supplementary Fig. 18). The more organic-rich SEI of the pristine Li foil is attributed to the surface inhomogeneity that locally builds strong reduction environment, which drives further reduction of metastable organic components, although an in-depth investigation is needed to elucidate the phenomenon in detail. This inconsistent SEI formation is linked with the dead Li that accumulates in conjunction with electrolyte decomposition^52^. The electrochemically active surface area for Li deposition could be inferred by measuring the exchange current (Fig. 7d and Supplementary Fig. 19). The exchange current density values of the naked and pristine Li foils were 0.61 and 0.06 mA cm^−2^ before cycling, respectively, and these values changed to 2.33 and 0.09 mA cm^−2^ after 100 cycles.

As it is well known that the dendritic growth is closely related to severe electrolyte decomposition that results in electrode swelling (Supplementary Fig. 16), the morphology of the Li deposits on the naked Li foil after cycling was further analyzed using high resolution X-ray tomography microscopy (XTM) (Fig. 7e and Supplementary Fig. 20). When reconstructed in 3D space (see details in Experimental), the columnar chunks (with each chunk sized 5−10 μm) were found to be interconnected and extended right through the entire layer of deposited Li with a thickness of 60 μm, implying that the majority of Li deposits remained electrically connected over 100 cycles, with a negligible amount of dead Li. From the perspective of the morphology, the fact that Li pillars (composed of chunks) and voids co-exist appears to be beneficial in terms of facilitating charge transfer and Li ion mass transfer. In fact, the electrical resistance between the outermost layer of deposited Li and the current collector reveals the electric connectivity of the Li deposits (Supplementary Fig. 21). After 100 cycles at 1 mA cm^−2^ and 1 mAh cm^−2^, the resistances of the pristine and naked Li cells differed by six orders of magnitude such that they had values of 16.35 MΩ and 2.6 Ω, respectively. As is clear from the insets in Supplementary Fig. 21, the glossy silver shade of the naked Li foil represents an active Li layer that is electrically connected through the layer forming the Li deposit.

The electrical connectivity and columnar morphology of the Li deposit has multiple advantages: (1) The overall current can be distributed through the Li columns such that the local current density decreases toward building lower polarization for Li plating (Supplementary Fig. 22). (2) The columnar morphology reduces the polarization relevant to Li ion mass transport through the inter-columnar voids. The mossy morphology, typically generated on the pristine Li foil, provides more tortuous pathways for Li^+^-ion transport, causing severe Li ion concentration gradients, which is usually reflected in the characteristic arcing shapes of its voltage profiles (Supplementary Fig. 23)^39^. On the other hand, in the case of the naked Li foil, the columnar layer lessens the Li ion concentration gradient and flattens the voltage profile shapes. The flat voltage profiles of high-concentration electrolytes that weaken the Li ion concentration gradient were similarly interpreted. (3) The growth of Li dendrites after a long cycling period under harsh operation conditions takes place mainly in the inter-columnar space (Supplementary Fig. 24), instead of on the topmost surface that produces a massive amount of dead Li near the separator. The direction of Li deposits is well known^53^ to affect the sustainability of cycling as well as the formation of short-circuits.

## Discussion

Most existing approaches designed to modify the Li foil for LMBs rely on the deposition of additional structures or protection layers on the native oxide layer. However, a passivation layer of even a few nanometers thick hinders uniform Li plating during the first charge, and uneven Li nucleation at this early stage can negatively affect the reversibility in subsequent cycles and ultimately the cycle life of the cell. Viewing this from the opposite direction, we removed the passivation layer by employing a simple bromine-based acid-base reaction that can be integrated with the cell assembly to prevent the Li foil from undergoing re-oxidation. The naked Li skin induces epitaxial Li plating in the initial cycle, markedly suppressing unwanted phenomena related to the native oxide layer such as non-uniform nucleation with a high overpotential. The marginal nucleation barrier on the naked Li skin drives the densely packed columnar morphologies of Li deposits, enabling stable cycling even at a high current density of 10 mA cm^−2^. Considering that the observed cell performance was enabled solely by removing the passivation layer, further improvement would be expected by incorporating other advanced approaches including electrolyte engineering and separator modifications.

## Methods

### Materials

Lithium nickel manganese cobalt oxide (LiNi_0.6_Co_0.2_Mn_0.2_O_2_ or NCM622, D50 = 10.7 μm, L&F corporation, South Korea) and LiFePO_4_ (LFP, ECOPRO BM, South Korea) were used for the cathodes. A solution of 1 M BBr_3_ in hexane, anhydrous DEC, anhydrous dimethyl carbonate (DMC), LiTFSI, LiFSI, LiNO_3_, anhydrous DOL, anhydrous DME, and anhydrous hexane were purchased from Sigma-Aldrich, USA. The electrolyte containing 1 M LiPF_6_ in EC/DEC (50/50 = v/v) with 10 wt% FEC was purchased from Wellcos Corporation, South Korea. Super P (Timcal, Switzerland) conductive carbon and poly(vinylidene fluoride) (PVDF, Kynar) binder were used for the NCM622 electrodes. N-methyl-2-pyrrolidone (NMP) was purchased from JUNSEI, Japan. Polyethylene (PE) separators (SK Innovation, South Korea) were used for coin-cell tests.

### Physicochemical characterization

FE-SEM images were acquired using a JSM-7600F instrument (JEOL, Japan). XPS results were recorded on an AXIS-His (KRATOS, U.K.) without exposure to air atmosphere. The BBr_3_-treated Li foil was washed with hexane to remove the residual BBr_3_ for LiBr characterization and was washed with 1 M LiPF_6_ in EC/DEC (50/50 = v/v) with 10 wt% FEC and with DEC to remove LiBr to characterize the naked Li surface. XRD analysis was performed in the 2θ range of 5° to 80° using a D8 Advance spectrometer (Bruker, Germany) from which ambient air was excluded. EBSD analysis was performed to determine the crystal orientation using an Oxford EBSD instrument (Oxford Instruments, U.K.) at the Research Institute of the Advanced Materials Research Center (RIAM) at Seoul National University. The electrical conductivity of the electrodeposited layer was measured in a glove box under Ar atmosphere using a digital multitester (HIOKI 3244-60, MISUMI, Japan). High resolution 3D XTM was performed to obtain 3D images of the electrodeposition layers using an Xradia 620 Versa instrument (Carl Zeiss, USA) at the National Centre for Inter-university Research Facilities (NCIRF) at Seoul National University. Li metal samples were sealed in airtight vials during the XTM analysis.

### Preparation of electrodes

To prepare naked Li foil, the punched Li foil was simply immersed in a 1 M BBr_3_ hexane solution at 60 °C for 3 h using a flask with an airtight cap. Well-controlled Ar atmosphere was required for the designated reaction. [Caution: BBr_3_ reacts strongly with various organic solvents to generate fumes.] After the color changed to light violet, the punched Li foil was washed several times with hexane to remove the residual BBr_3_. Subsequently, the process of removing the LiBr layer was performed by washing the foil with sufficient amounts of the conventional 1 M LiPF_6_ EC/DEC 10 wt% FEC electrolyte. After the LiBr layer was thoroughly washed with the running electrolyte, the cell was immediately assembled using the wet Li foil without a separate drying process to minimize the exposure to impurities in the glove box. For the full-cell tests, NCM622 electrodes were fabricated using NMP-based slurry. This slurry, consisting of NCM622, Super P, and PVDF binder in a weight ratio of 95:3:2, was cast on aluminum foil, followed by drying at 60 °C overnight under vacuum. The mass loading of NCM622 was 17.3 mg cm^−2^, corresponding to the areal capacity of 2.9 mAh cm^−2^ when measured at 0.1 C. LFP electrodes were fabricated in the same manner as their NCM622 counterparts. The weight ratio of LFP, Super P and PVDF binder was 90:5:5. The mass loading of LFP was 21.9 mg cm^−2^ and the areal capacity was fixed at 3.3 mAh cm^−2^.

### Electrochemical characterization

All electrochemical measurements were performed without rest period using CR2032 coin-type cells assembled in an Ar-filled glove box. All coin cells included a PE separator, and each cell contained 60 µL of the electrolyte. EIS measurements were performed using a potentiostat (VSP, Bio-Logic, France) over the frequency range from 1 MHz to 0.1 Hz. The symmetric cell tests were conducted using 1 M LiPF_6_ in EC/DEC (50/50 = v/v) with 10 wt% FEC electrolyte in most cases, and 5.5 M LiFSI DMC electrolyte was used for the high-concentration electrolyte test. All electrochemical cycling tests were performed in a chamber at 25 °C. The CV tests were conducted in the range of −200 mV to +200 mV to evaluate the nucleation barrier. Chronopotentiometry measurements were performed to measure the vulnerability to short-circuit formation at various current densities. The full-cell evaluations were conducted using NCM622 cathodes with an areal capacity of 2.9 mAh cm^−2^ or LFP cathodes with an areal capacity of 3.3 mAh cm^−2^. The NCM622 full-cell tests were carried out using 1 M LiPF_6_ in EC/DEC (50/50 = v/v) with 10 wt% FEC as electrolyte by charging at 0.5 C in CC-CV mode and discharging at 0.5 C in CC mode in the potential range of 2.7 − 4.3 V after pre-cycles of 0.1 C. The LFP full-cell tests were carried out using 1 M LiTFSI 0.2 M LiNO_3_ in DOL/DME (50/50 = v/v) as electrolyte by charging and discharging at 0.5 C in CC mode in the potential range of 2.5 − 4.0 V after pre-cycles of 0.1 C.

## Supplementary information

Supplementary Information

## Data Availability

The data that support the plots within this paper and other findings of this study are available from the corresponding author upon reasonable request.
