# Supplementary material for: Naked metallic skin for homo-epitaxial deposition in lithium metal batteries
Source: Nat Commun. 2023 Mar 9;14:1296. doi: 10.1038/s41467-023-36934-x (PMC9998607; doi:10.1038/s41467-023-36934-x)
Supplement: Supplementary file 1 — Supplementary Information [file 41467_2023_36934_MOESM1_ESM.pdf]

Naked metallic skin for homo-epitaxial deposition in lithium metal batteries

Minsung Baek[1,6], Jinyoung Kim[1,6], Kwanghoon Jeong[2], Seonmo Yang[1], Heejin Kim[3], Jimin Lee[1], Minkwan Kim[1], Ki Jae Kim[4]* and Jang Wook Choi[1,5]*

[1]School of Chemical and Biological Engineering and Institute of Chemical Processes, Seoul National University, Seoul, Republic of Korea.

[2]Department of Chemistry, Seoul National University, Seoul, Republic of Korea.

[3]Electron Microscopy Research Center, Korea Basic Science Institute, Daejeon, Republic of Korea

[4]Department of Energy Engineering, Konkuk University, Seoul, Republic of Korea

[5]Department of Materials Science and Engineering, Seoul National University, Seoul, Republic of Korea.

[6]These authors contributed equally: Minsung Baek, Jinyoung Kim.

*E-mail: jangwookchoi@snu.ac.kr

Supplementary Figures

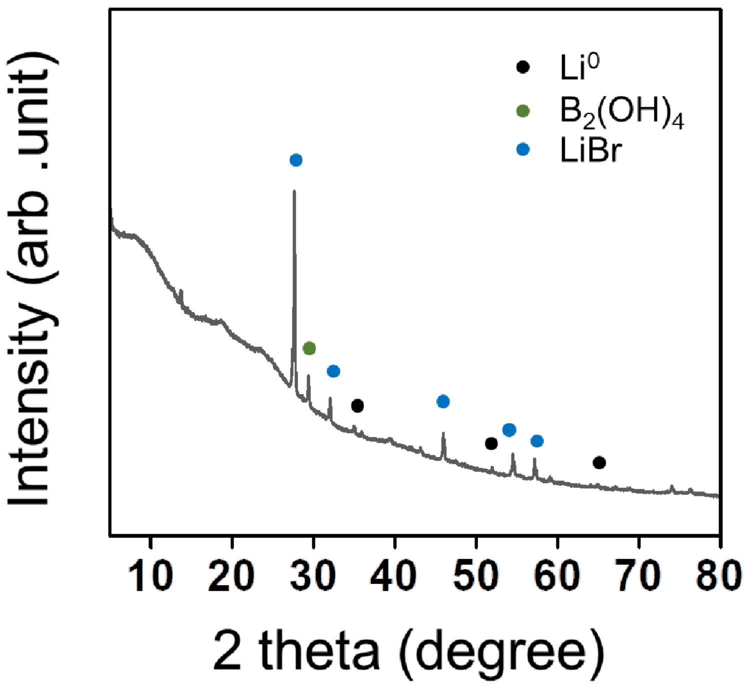

Supplementary Figure 1 | XRD patterns of the Li metal foil after BBr_3 treatment for 1 week followed by washing with hexane. $B_2(OH)_4$ is predicted to be the oxidized form of B_2Br_4.

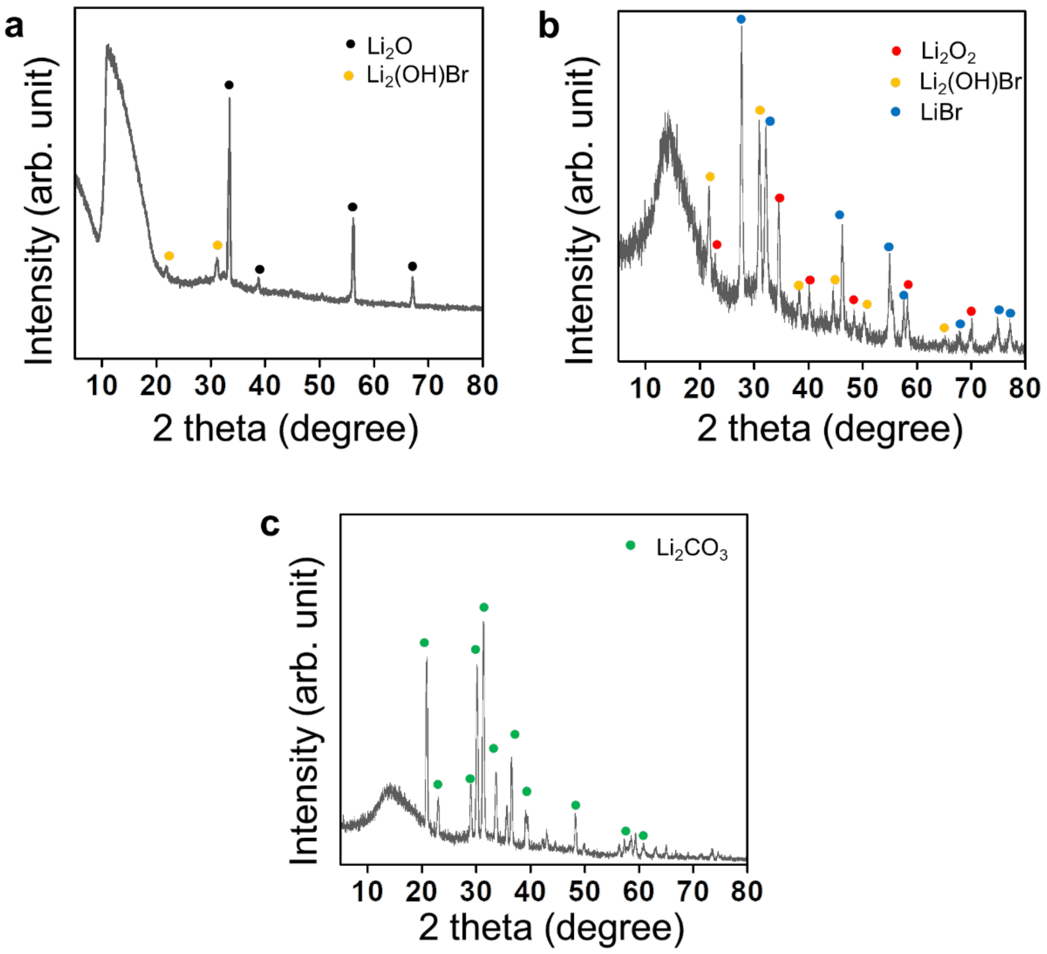

Supplementary Figure 2 | XRD patterns of **a**, Li_2O, **b**, Li_2O_2, **c**, Li_2CO_3 powders after treatment with BBr_3 for 1 week followed by washing with hexane.

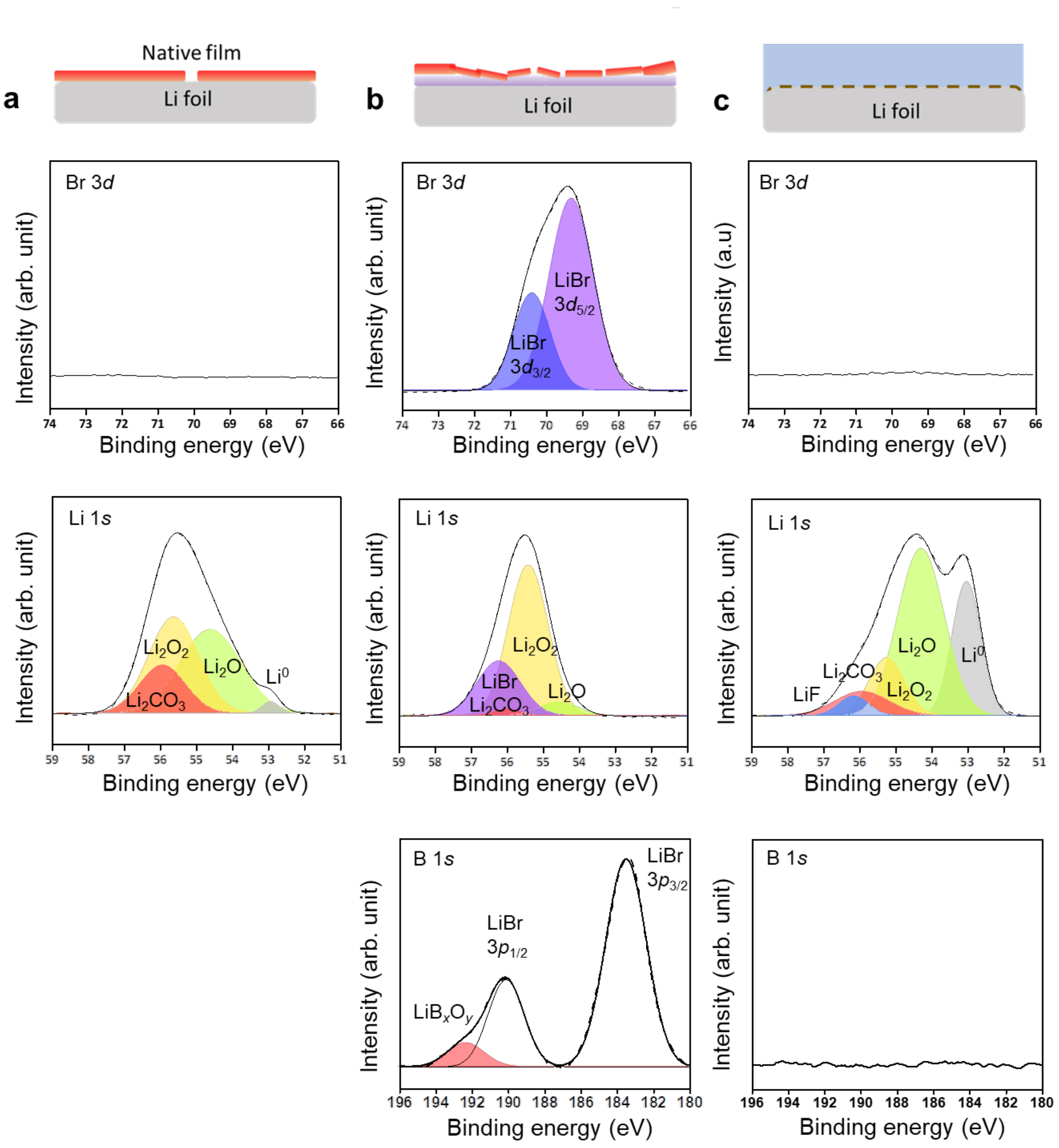

Supplementary Figure 3 | XPS profiles of **a**, the pristine Li metal foil, **b**, the BBr_3-treated Li metal foil after washing with hexane, and **c**, the BBr_3-treated Li metal foil after washing with hexane, electrolyte, and DEC in sequence.

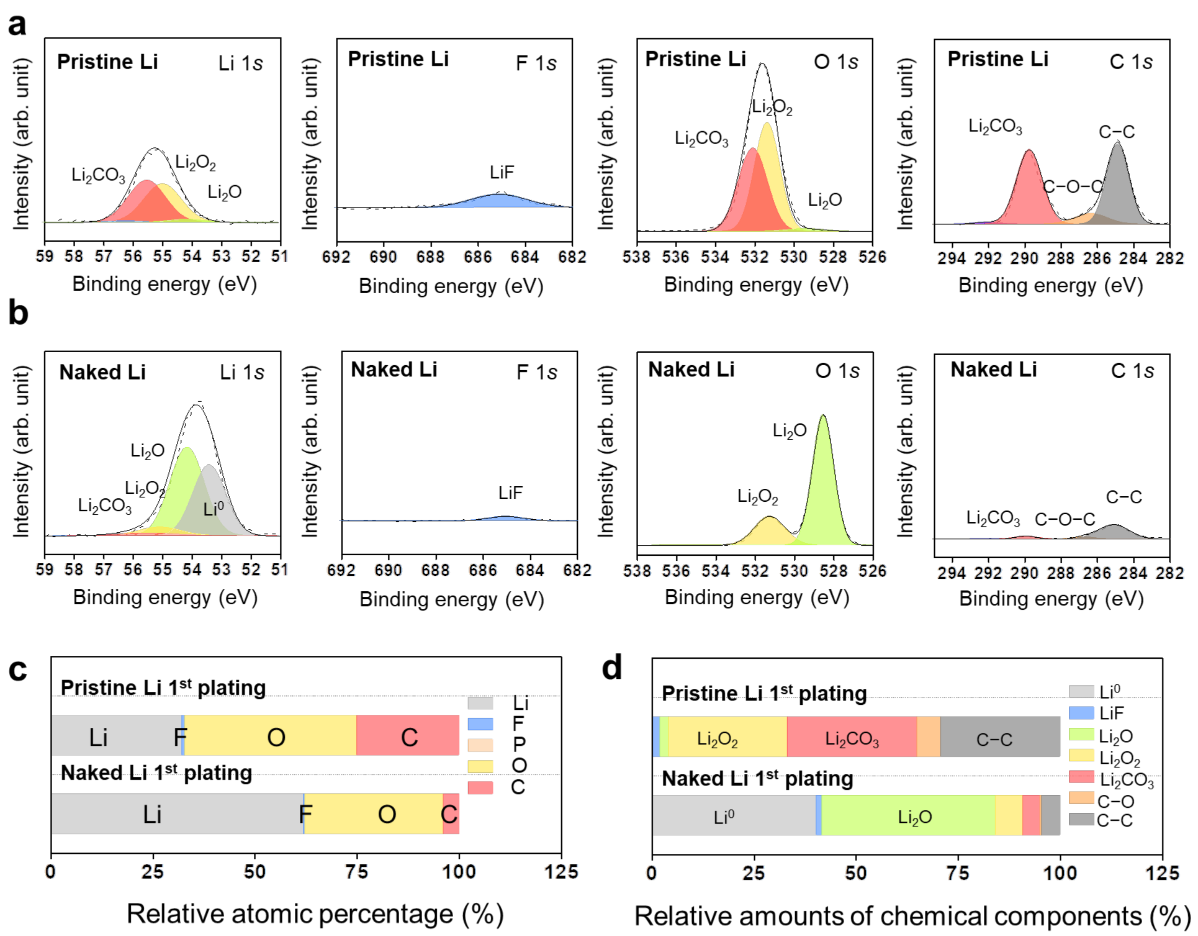

Supplementary Figure 4 | XPS results on the SEI components of **a**, pristine Li and **b**, naked Li after the 1st electrodeposition. **c,** Relative atomic percentage and **d,** the relative amounts of chemical components in the SEI of the pristine and naked Li after the 1st electrodeposition.

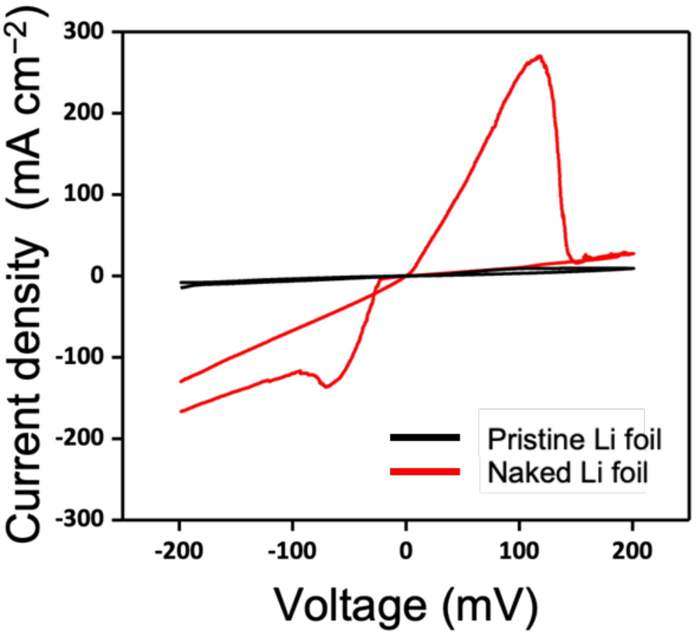

Supplementary Figure 5 | CV profiles of the pristine (black) and naked (red) Li-Li symmetric cells in the range of −200 mV to +200 mV.

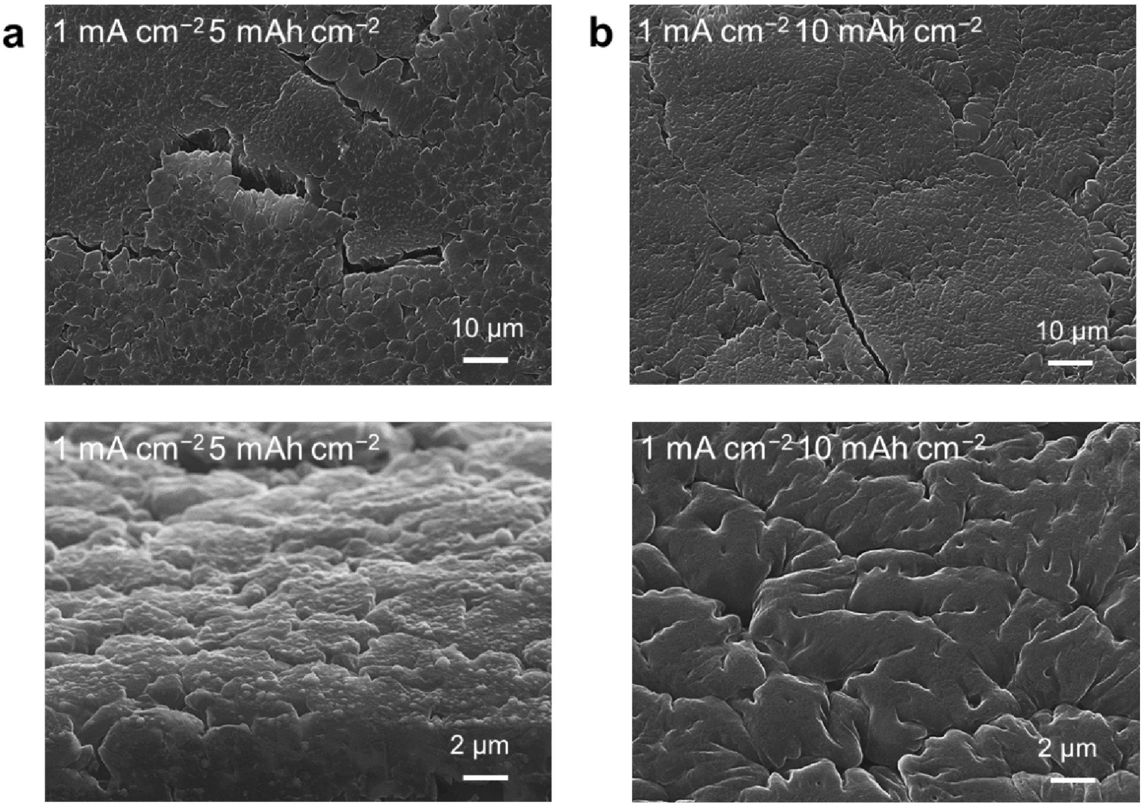

Supplementary Figure 6 | a, SEM images of the Li deposits on the naked Li foil after 5 mAh cm^{-2} deposition at the current density of 1 mA cm^{-2}. **b**, SEM images of the Li deposits on the naked Li foil after 10 mAh cm^{-2} deposition at the current density of 1 mA cm^{-2}.

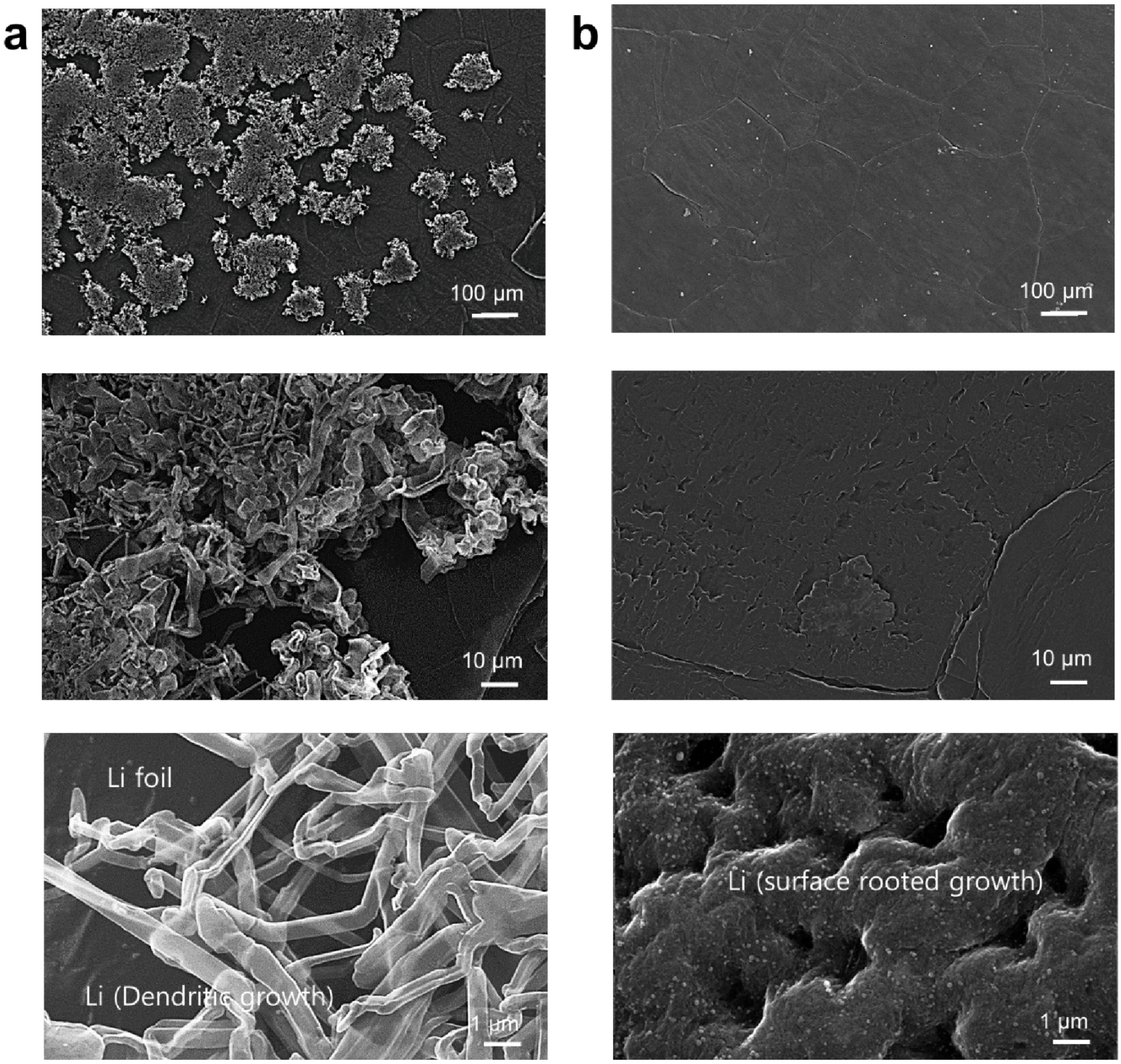

Supplementary Figure 7 | SEM images of the Li deposits on **a**, the pristine and **b**, the naked Li foil after 1 mAh cm^{-2} deposition at the current density of 1 mA cm^{-2}.

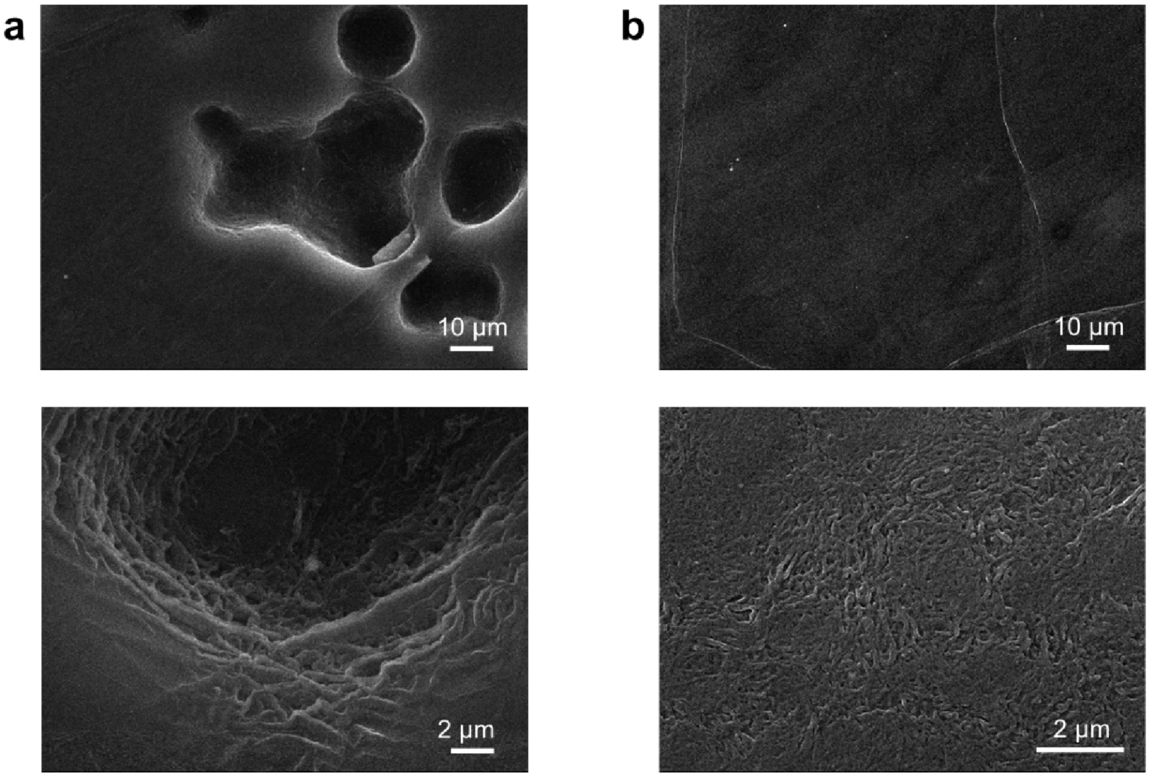

Supplementary Figure 8 | SEM images of the pit formation on **a**, the pristine and **b**, the naked Li foil after 1 mAh cm^{-2} stripping at the current density of 1 mA cm^{-2}.

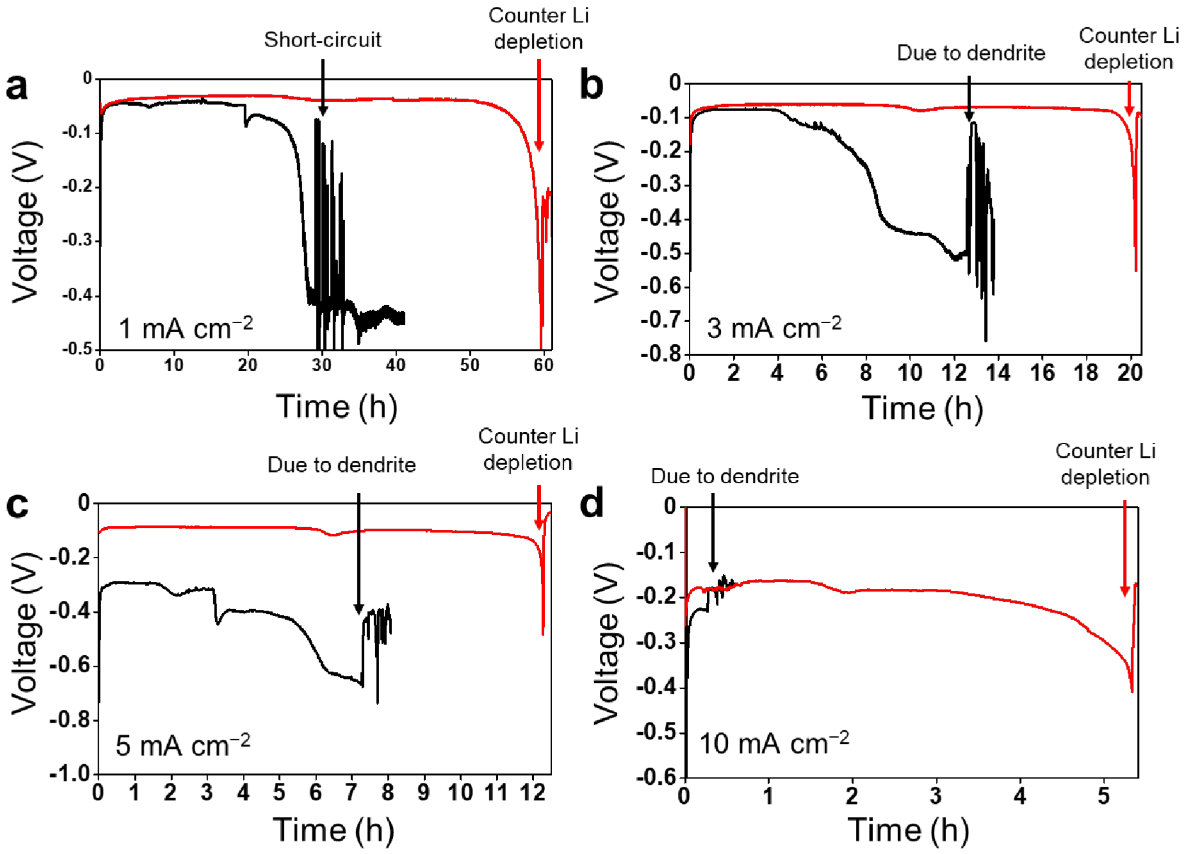

Supplementary Figure 9 | Chronopotentiometry measurements of the pristine (black) and naked (red) Li-Li symmetric cells at a current density of **a**, 1 mA cm^{-2}, **b**, 3 mA cm^{-2}, **c**, 5 mA cm^{-2}, and **d**, 10 mA cm^{-2}. The thicknesses of the working and counter Li foils were both 300 μm.

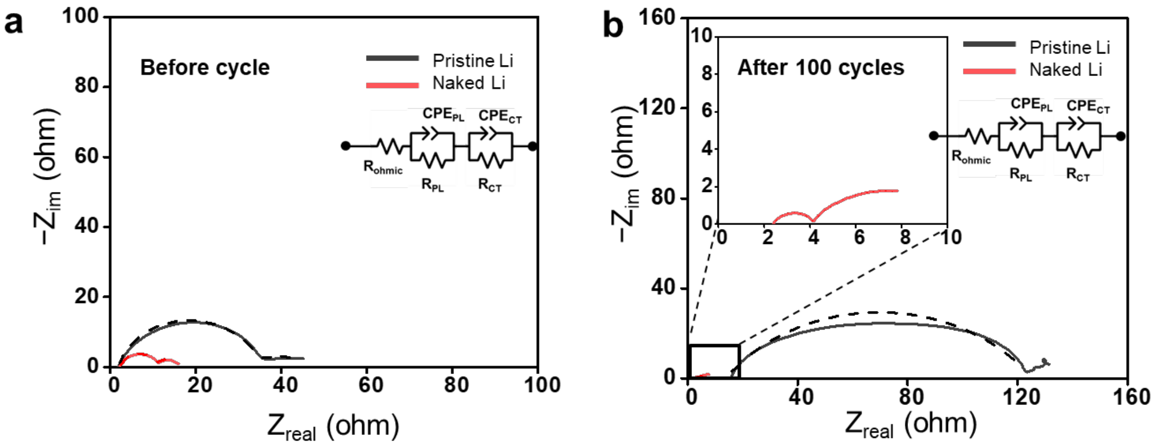

Supplementary Figure 10 | Nyquist of the pristine and naked Li-Li symmetric cells **a**, before and **b,** after 100 cycles. In the case of the pristine cell, the formation of soft short-circuits severely deforms the charge transfer resistance at low frequency.

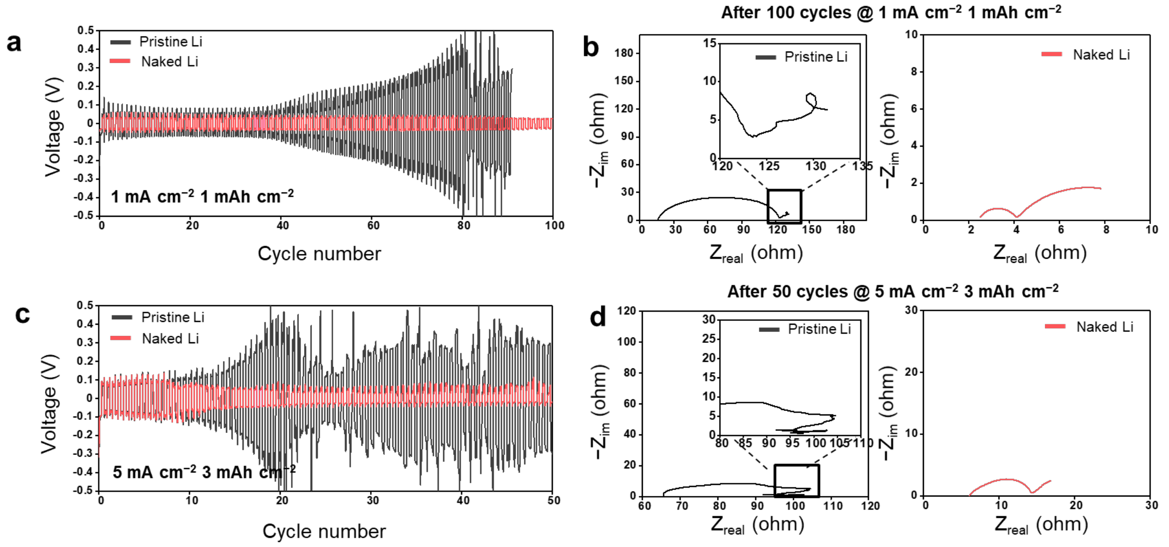

Supplementary Figure 11 | a, Voltage profiles of the pristine and naked Li-Li symmetric cells at 1 mA cm^{-2} 1 mAh cm^{-2}. **b**, EIS results of the pristine and naked Li-Li symmetric cells after 100 cycles at 1 mA cm^{-2} 1 mAh cm^{-2}. **c**, Voltage profiles of the pristine and naked Li-Li symmetric cells at 5 mA cm^{-2} 3 mAh cm^{-2}. **d**, EIS results of the pristine and naked Li-Li symmetric cells after 50 cycles at 5 mA cm^{-2} 3 mAh cm^{-2}. 1 M $LiPF_6$ in EC/DEC (50/50=v/v) 10% FEC electrolyte was used for the tests.

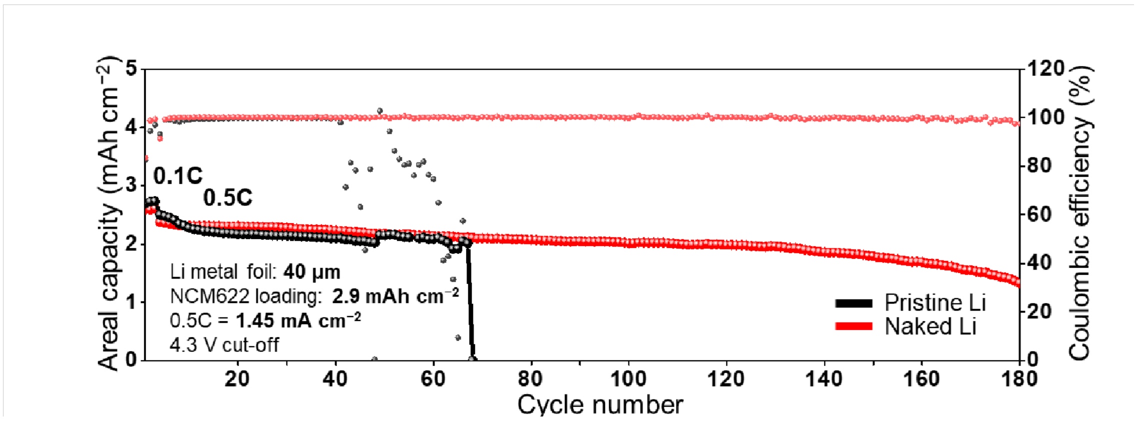

Supplementary Figure 12 | Discharge capacity and Coulombic efficiency against the cycle number of full cells with 2.9 mAh cm^{-2} NCM622 as cathode and 40 µm pristine (black) and naked (red) Li metal as anodes. 1 M $LiPF_6$ in EC/DEC (50/50=v/v) 10% FEC was used for the tests.

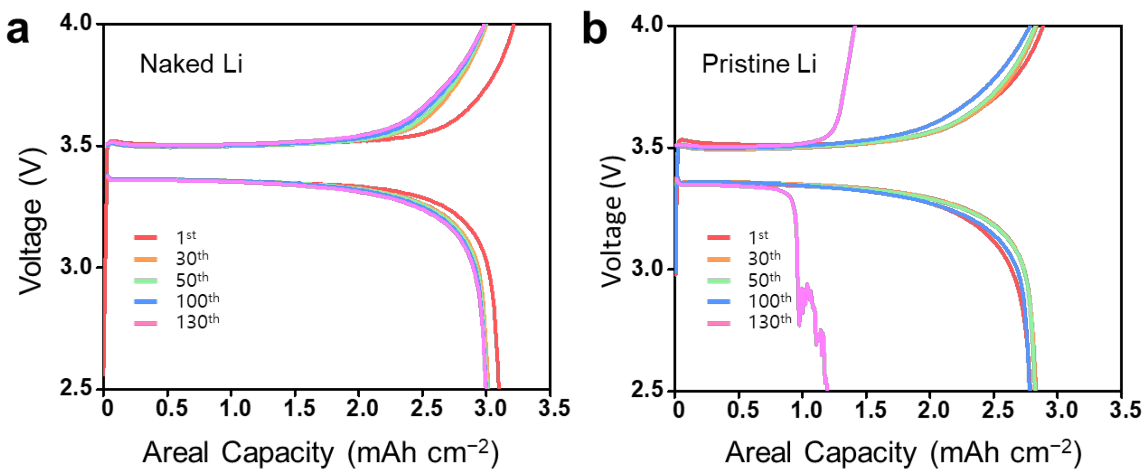

Supplementary Figure 13 | Galvanostatic potential profiles of the full cells fabricated with 3.3 mAh cm^{-2} LFP as cathode and 40 μm naked (left) and pristine (left) Li metal foils as anodes for a different number of cycles.

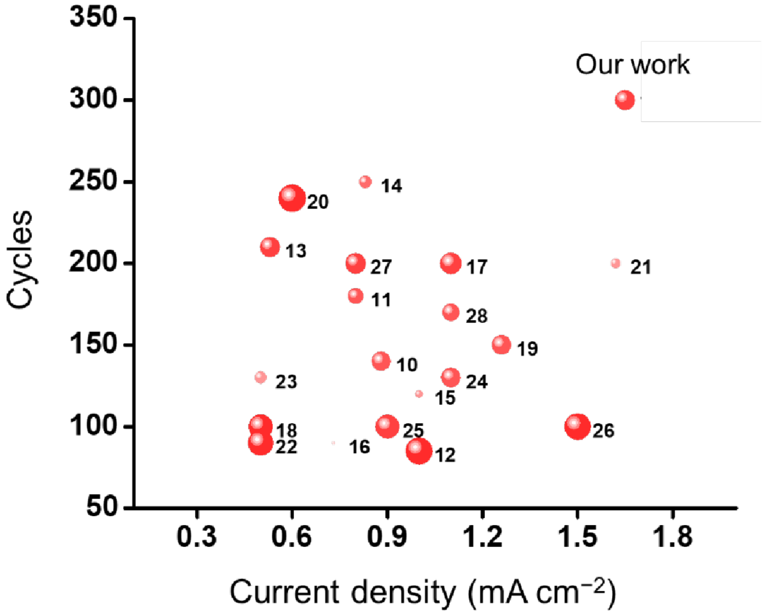

Supplementary Figure 14 | Cycle performance at various current densities compared to literature with limited amount of lithium metal corresponding to an N/P ratio less than 4, shown in Table S2. The size of the circle reflects the N/P ratio.

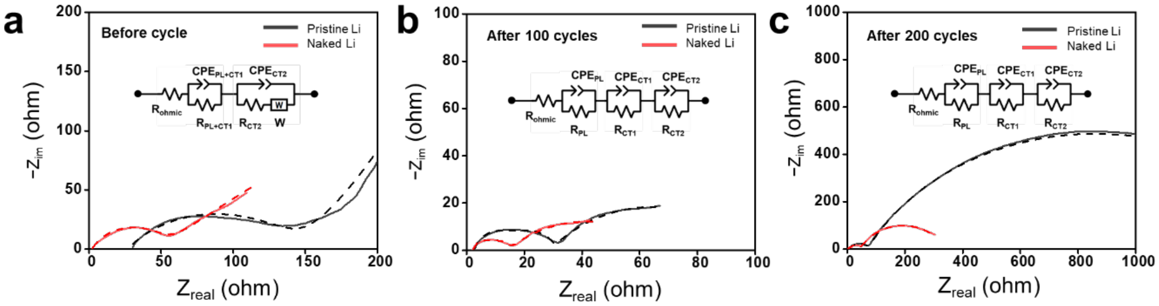

Supplementary Figure 15 | EIS measurements of the full cells containing 3.3 mAh cm^{-2} LFP as cathode and 40 µm-thick pristine (black) or naked (red) Li metal foil as anode **a**, before cycling, **b**, after 100 cycles, and **c,** after 200 cycles.

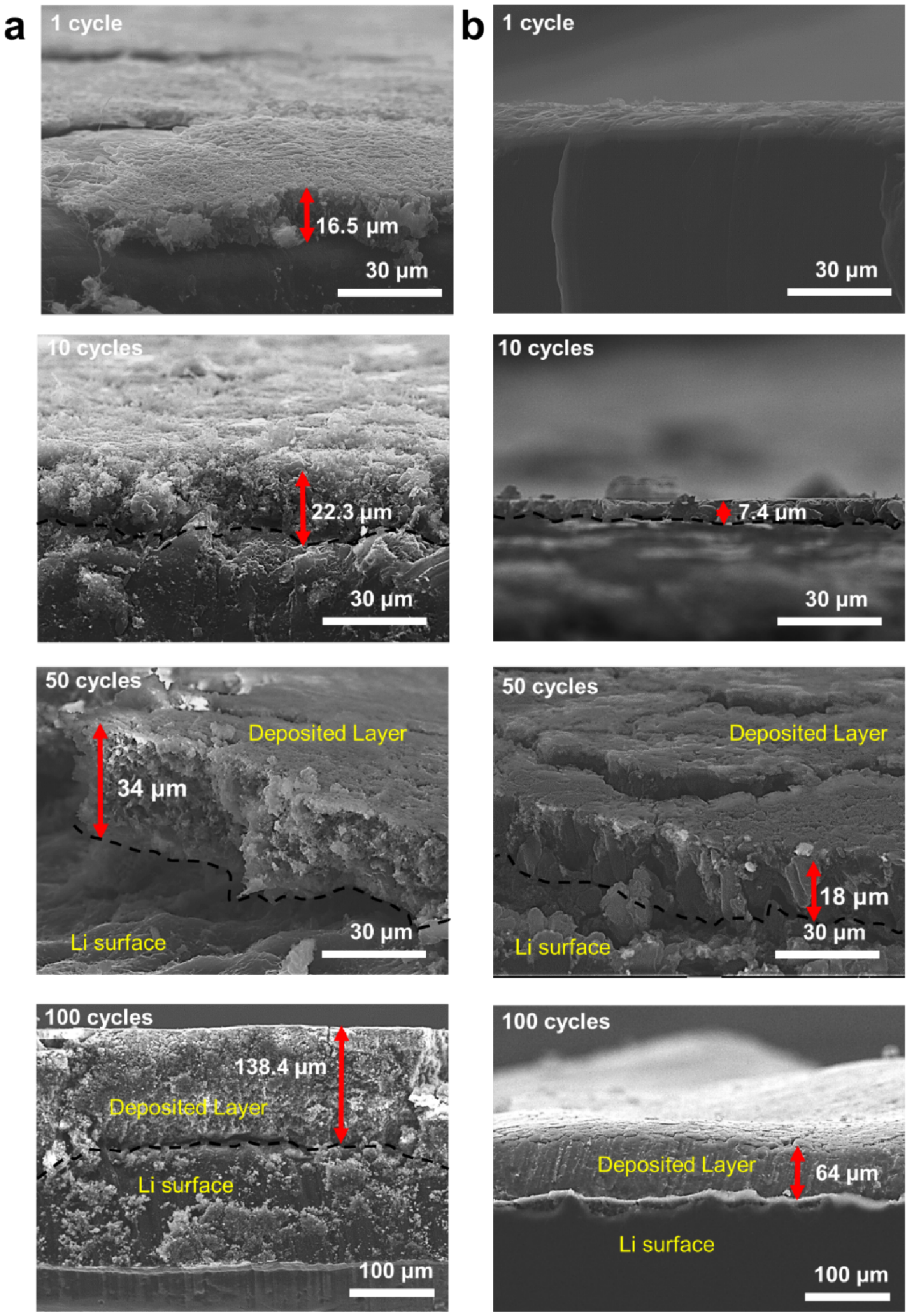

Supplementary Figure 16 | Cross-sectional SEM images of the Li deposit layers of **a**, the pristine and **b**, the naked Li-Li symmetric cells after 1, 10, 50, and 100 galvanostatic cycles at 1 mA cm^{-2} with the specific capacity of 1 mAh cm^{-2}.

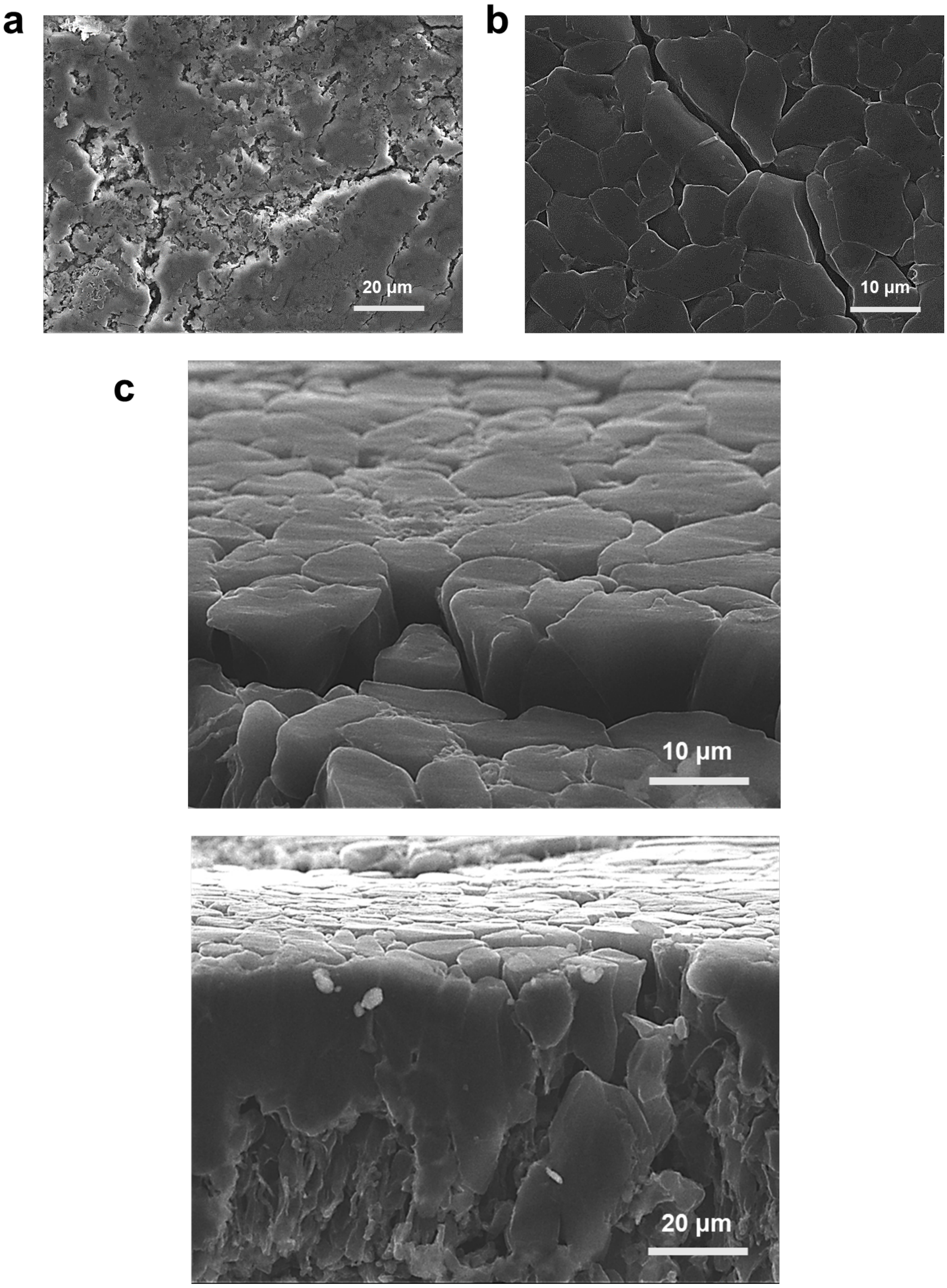

Supplementary Figure 17 | SEM images of the Li deposits on the **a**, pristine and **b**, naked Li foil after 100 galvanostatic cycles at 1 mA cm^{-2} with the specific capacity of 1 mAh cm^{-2}. **c**, Tilted SEM images of the Li deposits on the naked Li foil after the same number of cycles under the same conditions.

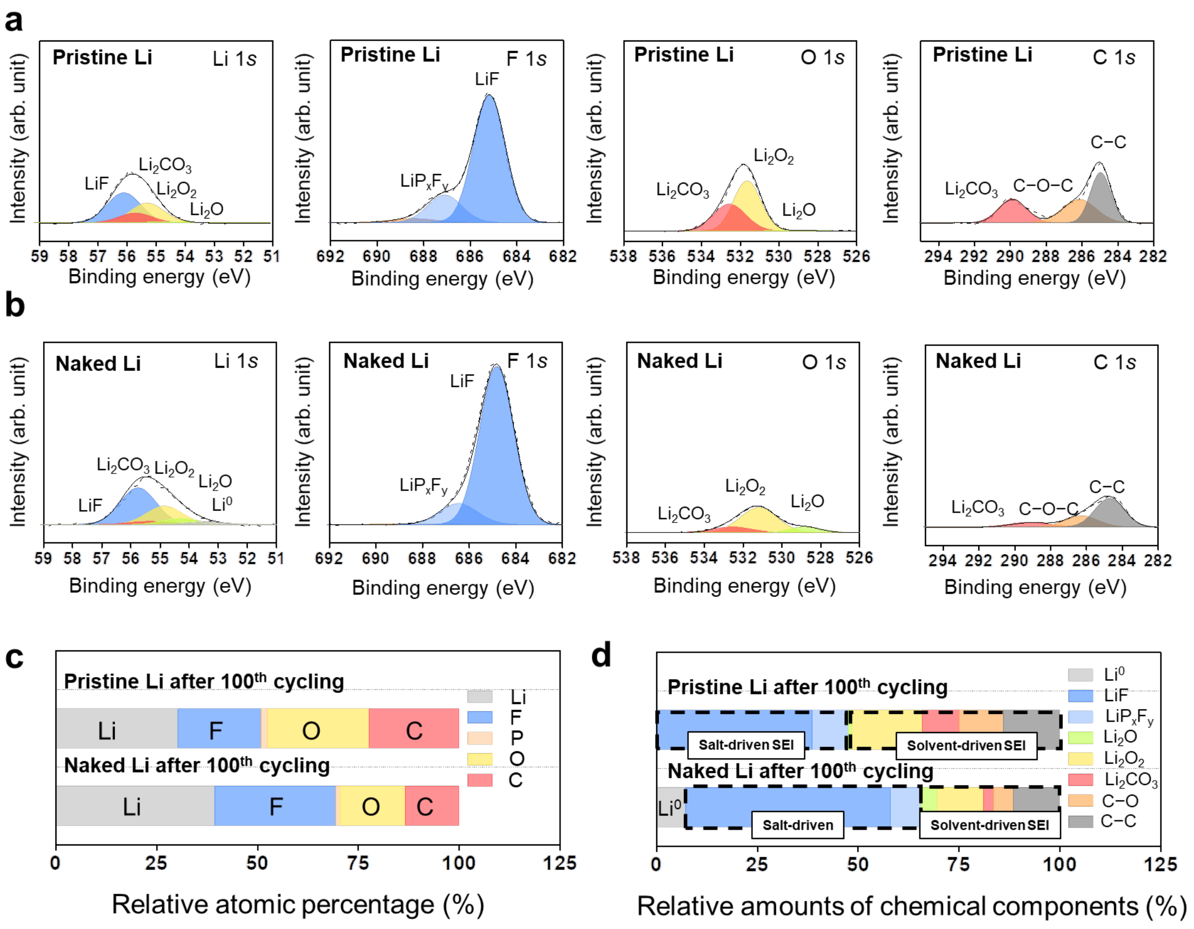

Supplementary Figure 18 | XPS results on the SEI components of **a**, pristine Li and **b**, naked Li after 100 cycles. **c,** Relative atomic percentage and **d,** the relative amounts of chemical components in the SEI of the pristine and naked Li after 100 cycles.

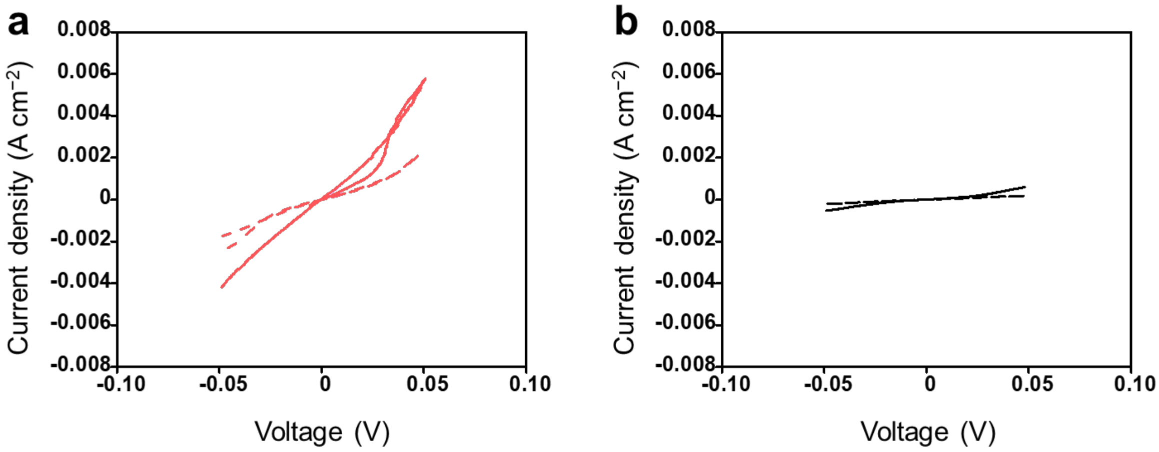

Supplementary Figure 19 | CV profiles of the **a**, naked and **b**, pristine Li-Li symmetric cells within the potential range of −50 mV to +50 mV before (dashed line) and after (solid line) 100 galvanostatic cycles at 1 mA cm^{-2} with the specific capacity of 1 mAh cm^{-2}.

a

b

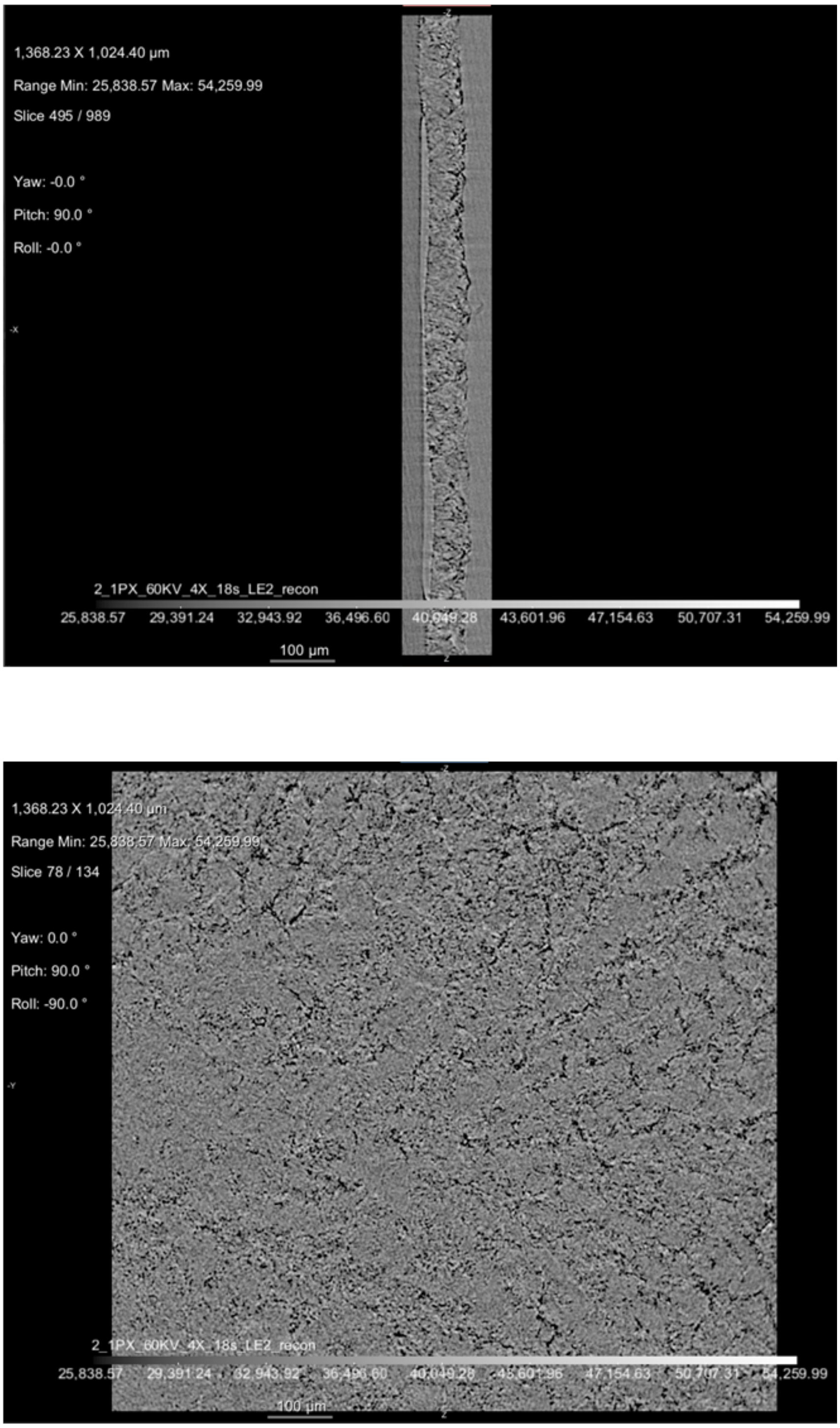

Supplementary Figure 20 | XTM image of the naked Li foil after 100 cycles at 1 mA cm^{-2} with the specific capacity of 1 mAh cm^{-2}: **a**, cross-sectional and **b**, top views.

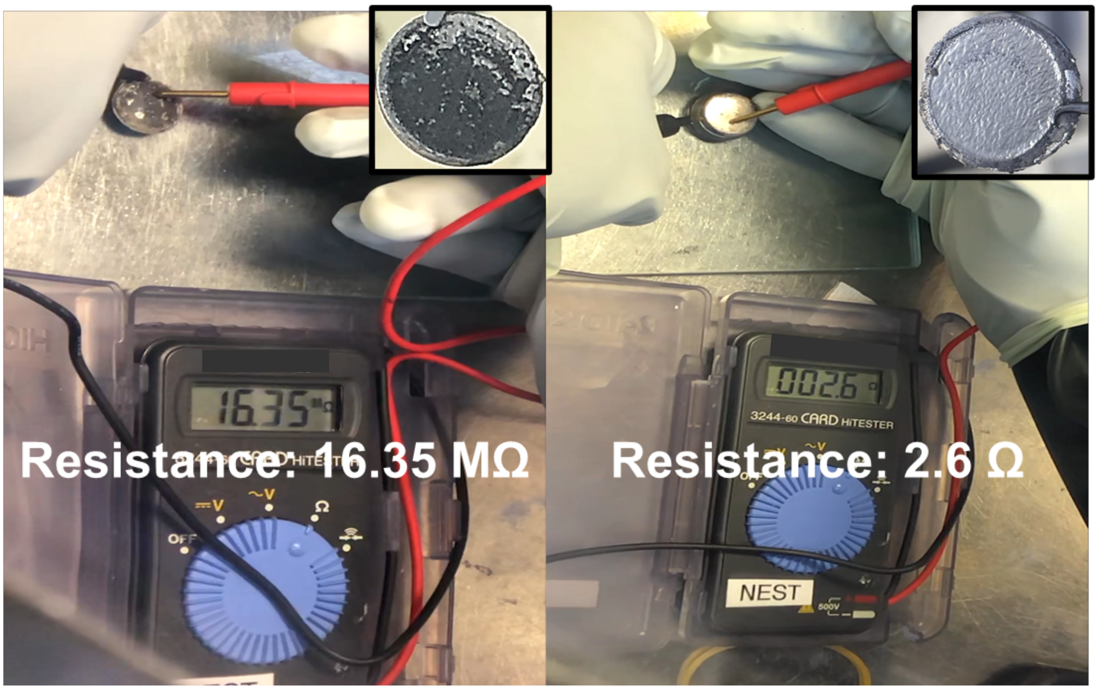

Supplementary Figure 21 | Electrical resistance measurements between the Li deposit layer and the current collector for the pristine (left) and naked Li foils (right) after 100 cycles at 1 mA cm^{-2} with the specific capacity of 1 mAh cm^{-2}. Insets are the photographs of the corresponding Li foils.

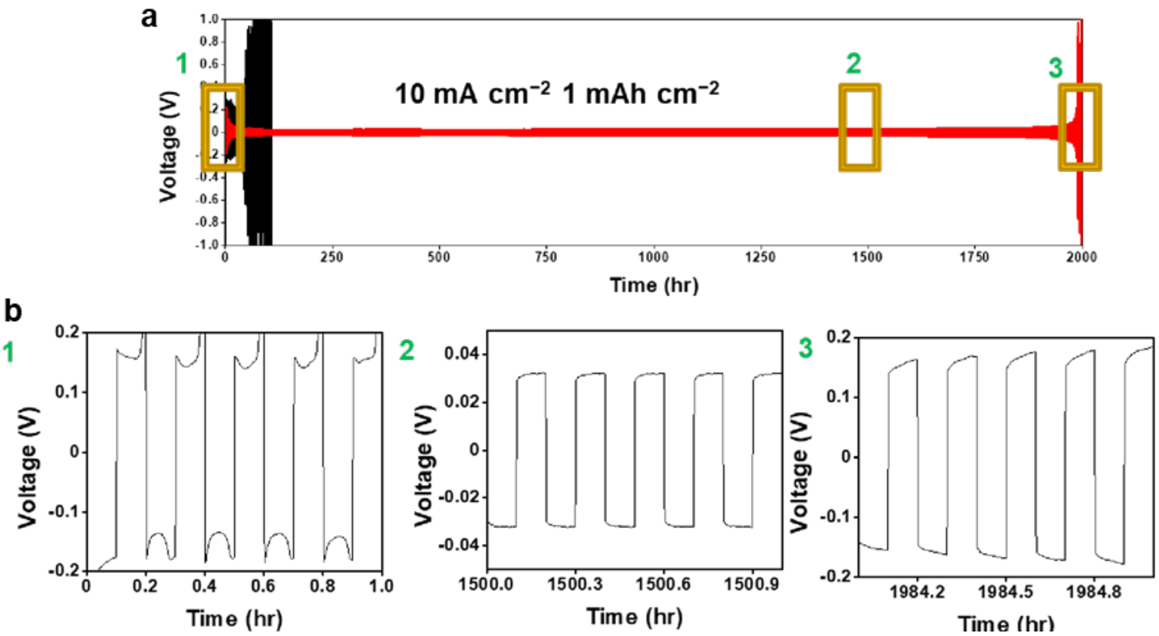

Supplementary Figure 22 | a, Galvanostatic charge-discharge voltage profiles of the pristine (black) and naked (red) Li−Li symmetric cells when operated at 10 mA cm^{-2} with the specific capacity of 1 mAh cm^{-2}. **b**, Magnified voltage profiles of the naked Li-Li symmetric cell at different time ranges.

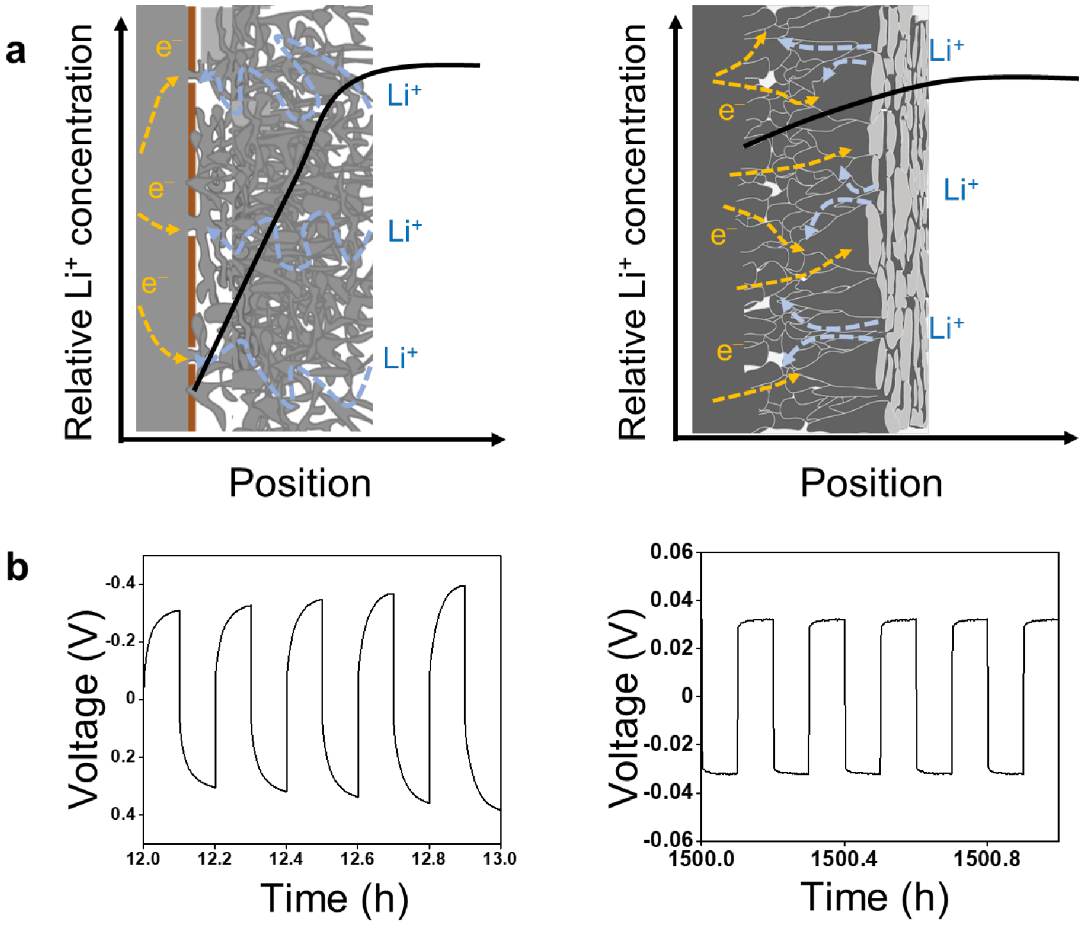

Supplementary Figure 23 | a, Schematic illustrations of the concentration gradient under the mass-transfer limited regime shortly before cell failure of the pristine (left) and naked (right) Li-Li symmetric cells. **b**, Voltage profiles of the (left) pristine and (right) naked Li-Li symmetric cells during cycling at 10 mA cm^{-2} with the specific capacity of 1 mAh cm^{-2}.

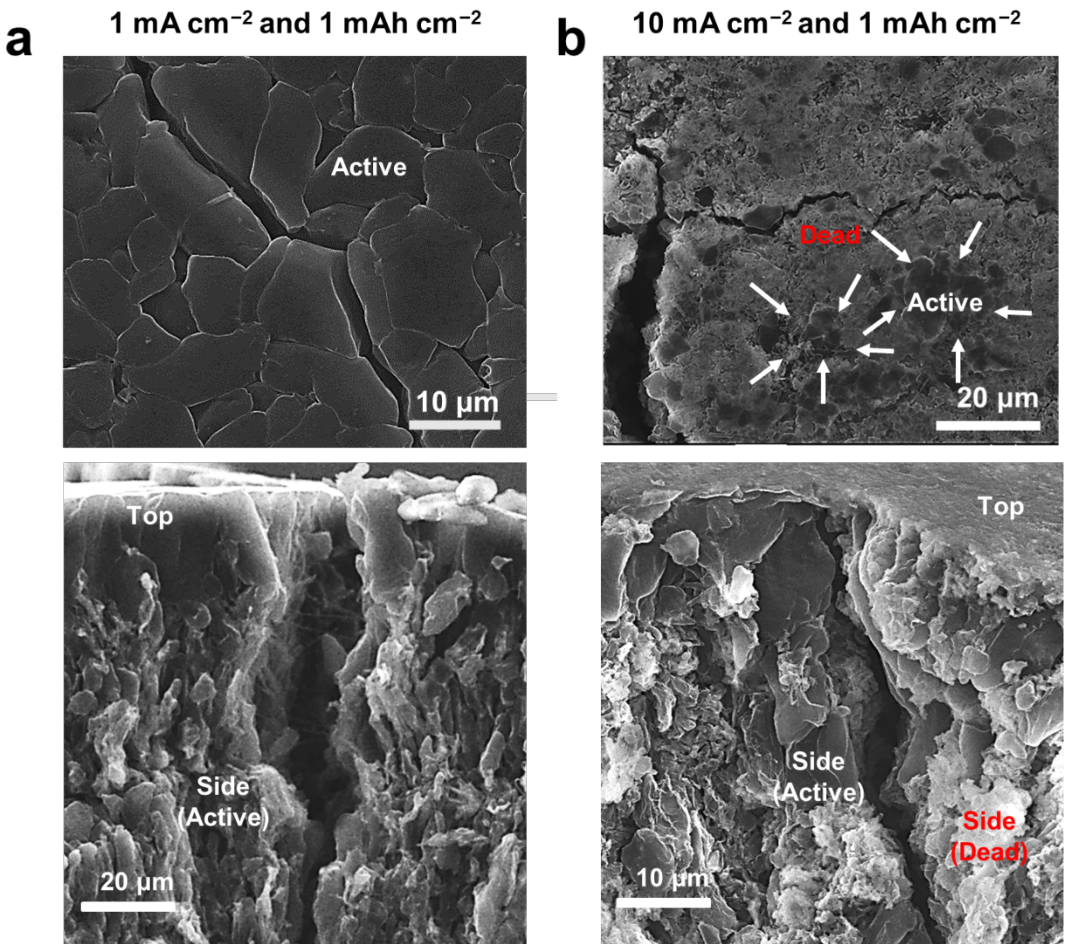

Supplementary Figure 24 | SEM images of the Li deposit layers on the naked Li foil. **a**, After 100 cycles at 1 mA cm^{-2} and 1 mAh cm^{-2}. **b**, When the cell fails after 10,000 cycles at 10 mA cm^{-2} and 1 mAh cm^{-2}. (Top) Top views. (Bottom) Cross-sectional views focusing on the bottom Li deposition layers.

Supplementary Tables

	Before cycle		After 100 cycles	
	Pristine	Naked	Pristine	Naked
Ohmic resistance	1.98 Ω	2.37 Ω	13.87 Ω	2.44 Ω
Passivation layer resistance	11.44 Ω	6.53 Ω	111.5 Ω	1.63 Ω
Anode charge-transfer resistance	32.46 Ω	18 Ω	-	6.33 Ω

Supplementary Table 1 | Fitting of the EIS results of the pristine and naked Li-Li symmetric cells before and after 100 cycles.

**Approach**	**Electrolyte**	**Current density [mA cm^{-2}]**	**Areal capacity [mAh cm^{-2}]**	**Short-circuit time [hours]**	**Reference**
ZnO-coated polyimide matrix	1 M $LiPF_6$ in EC/DEC (50/50=v/v)	3	1	67	S1
Si-coated carbon fiber matrix	1 M $LiPF_6$ in EC/DEC (50/50=v/v)	3	1	85	S2
Graphene matrix	1 M $LiPF_6$ in EC/DEC (50/50=v/v) 2% VC[a)]	3	1	69	S3
Carbonized wood conductive Li matrix	1 M $LiPF_6$ in EC/DEC (50/50=v/v)	3	1	150	S4
Naked Li	1 M $LiPF_6$ in EC/DEC (50/50=v/v) 10% FEC	3	3	1300	Our work
Pretreatment of the Li–metal anode with $LiNO_3$ adds a prior Li_2O-rich SEI layer	1 M $LiPF_6$ 0.05 M LiDFOB[b)] in EMC/FEC (75/25=v/v)	3.6	1.8	200	S5
Li_xSi-Li_2O matrix	1 M $LiPF_6$ in EC/DEC (50/50=v/v) 10% FEC 1% VC	10	1	20	S6
Fibrous metal felt as Li matrix	1 M $LiPF_6$ in EC/DEC (50/50=v/v)	10	10	200	S7
Few-micrometersthick Nafion protective layer	1 M $LiPF_6$ in EC/DEC (50/50=v/v)	10	40	2000	S8
Mesoporous AlF_3 framework	1 M $LiPF_6$ in EC/DEC (50/50=v/v) 10% FEC 1% VC	10	1	194	S9
Naked Li	1 M $LiPF_6$ in EC/DEC (50/50=v/v) 10% FEC	10^2	1	2000	Our work

[a)]Vinylene carbonate;

[b)]Lithium difluoro(oxalato)borate

Supplementary Table 2 | Comparison of short-circuit formation time at high current density in carbonate electrolyte-based 3D host studies.

Approach	Electrolyte	Current density [mA cm^{-2}]	Anode [µm] │Cathode [mAh cm^{-2}] │ N/P ratio	Capacity retention	Reference
SL[a)]-based HCE[b)] + 3D polyimide	1:2.5 LiFSI/SL	0.88	Li (20) l LFP (1.76) l 2.34	140, 92.7%	S10
DEE[c)]-based HCE	4 M LiFSI in DEE	0.8	Li (50) l NMC811 (4.8) l 2	180, 80%	S11
EC/EMC-based LHCE[d)] +LiDFOB[e)]	1.2 M LiFSI + 0.15 M LiDFOB in EC-EMC-BTFE[f)] (0.22:0.88:2.2)	1	Li (50) l NMC811 (3) l 3.33	85, 80%	S12
Fluorinated etherbased WSE[g)]	1 M LiFSI in FDMB[h)]	0.53	Li (20) l NMC523 (1.6) l 2.5	210, 99.3%	S13
Fluorinated etherbased WSE	1 M LiFSI in DME-FDMH[i)] (1/6=v/v)	0.83	Li (20) l NMC532 (2.5) l 1.6	250, 84%	S14
Fluorinated etherbased WSE	1 M LiPF$_6$ in FEC-FEMC[j)]-HFE[k)] (2:6:2 by wt%)	1	Li (9.7) l NMC811 (2) l 1	120, 95%	S15
Sulfonamide-based electrolyte	1 M LiFSI/DMTMSA[l)]	0.73	Li (9.2) l NMC811 (4.86) l 0.39	90, 88%	S16
Free-standing hollow carbon host	1 M LiTFSI in DOL/DME (1:1 w/w) with 1 wt% LiNO$_3$	1.1	Carbonized cotton (6 mAh cm^{-2}) l LFP (2.21) l 2.71	200, 91.3%	S17
3D copper host	1 M LiTFSI in DOL/DME (1:1 w/w) with 1 wt% LiNO$_3$	0.5	Porous Cu (3.0 mAh cm^{-2} l LFP (0. 0.986) l 3.04	100, 90%	S18
Ether-based LHCE	LiFSI-1.2DME-3TTE[m)]	1.26	Li (50) l NMC811 (4.2) l 2.45	150, 80%	S19
MOF[n)] nanocapsules additive	1 M LiPF$_6$ in EC/DEC (50/50=v/v) + LiNO$_3$@MOF 100 mg mL^{-1}	0.6	Li (50) l LCO (3) l 3.43	240, 90%	S20
Free-standing rGO carbon host	1 M LiPF$_6$ in EC/DEC (50/50=v/v) +1% VC + 10% FEC	1.62	Li@eGF (4 mAh cm^{-2}) l LFP (3.24) l 1.23	200, 81%	S21
FEC-based electrolytes	1.0 M LiPF$_6$ in FEC/DMC (1:4)	0.5	Li (50) l NMC622 (3.3) l 3.13	90, 90%	S22
FEC-based HCE	7.0 M LiFSI in FEC	0.5	Li (13) l LiNi$_{0.5}$Mn$_{1.5}$O$_4$ (1.8) l 1.49	130, 80%	S23
Trifluoroacetate (TFA) salt based electrolyte	1.0 M LiTFA in DME/FEC	1.1	Li (13) l NMC622 (1.1) l 2.44	130, 86%	S24
Carbonate-based HCE +LiDFOB	4.0 M LiTFSI/0.5 M LiDFOB in FEC/DMC	0.9	Li (26) l LiNi$_{0.5}$Mn$_{1.5}$O$_4$ (1.8) l 2.98	100, 88%	S25
Fluorinated diluent additive	1.3 M LiFSI/0.7 M LiTFSI/0.02 M LiPF$_6$ in DMC/1,2- dfBen[o)]	1.5	Li (40) l NMC523 (2.5) l 3.3	100, 90%	S26

Various salt based electrolyte	0.06 M Al(OTf)$_3$[p)]+0.5 M $LiNO_3$+0.2 M LiDFOB+0.2 M LiBOB[q)]+0.6 M LiFSI+0.05 M $LiPF_6$+0.03	0.8	Li (50) l NMC811 (4) l 2.57	200, 92.5%	S27
	M $LiBF_4$ in EC/DMC (2/1=v/v)				
Solubilizer mediated conventional carbonate electrolyte	1 M $LiPF_6$ in EC/DEC + 10 mM In(OTf)$_3$ + 0.5 M $LiNO_3$	1.1	Li (45) l NMC811 (4.3) l 2.14	170, 80%	S28
Naked Li	1 M LiTFSI 0.2 M $LiNO_3$ in DOL/DME (50/50=v/v)	1.65	Li (40) l LFP (3.3) l 2.5	300, 86.1%	Our work

[a)]Sulfolane;

[b)]Highly concentrated electrolyte;

[c)]1,2-diethoxyethane;

[d)]Localized high concentration electrolyte;

[e)]Lithium difluoro(oxalato)borate;

[f)]Bis(2,2,2-trifluoroethyl) ether;

[g)]Weakly solvated electrolyte;

[h)]Fluorinated 1,4-dimethoxylbutane;

[i)]Fluorinated 1,6-dimethoxyhexane;

[j)]3,3,3-fluoroethylmethyl carbonate;

[k)]1,1,2,2-tetrafluoroethyl-2′,2′,2′-trifluoroethyl ether;

[l)]N,N-dimethyltrifluoromethane-sulfonamide;

[m)]1,1,2,2-tetrafluoroethyl-2,2,3,3-tetrafluoropropyl ether;

[n)]Metal-organic framework;

[o)]1,2-difluoro benzene;

[p)]Trifluoromethanesulfonate;

[q)]Lithium Bis(oxalate) Borate;

Supplementary Table 3 | Comparison of capacity retention at various current density under a limited amount of lithium metal source with an N/P ratio less than 4.

<table>
<tr><th></th><th colspan="2">Before cycles</th><th colspan="2">After 100 cycles</th><th colspan="2">After 200 cycles</th></tr>
<tr><th></th><th>Pristine</th><th>Naked</th><th>Pristine</th><th>Naked</th><th>Pristine</th><th>Naked</th></tr>
<tr><td>Ohmic resistance</td><td>26.7 Ω</td><td>2.1 Ω</td><td>2.8 Ω</td><td>2.3 Ω</td><td>4.5 Ω</td><td>2.5 Ω</td></tr>
<tr><td>Passivation layer resistance</td><td rowspan="2">115.9 Ω</td><td rowspan="2">48.2 Ω</td><td>11.7 Ω</td><td>6.7 Ω</td><td>65.7 Ω</td><td>47.3 Ω</td></tr>
<tr><td>Anode charge-transfer resistance</td><td>72.3 Ω</td><td>54.7 Ω</td><td>1573 Ω</td><td>287.1 Ω</td></tr>
<tr><td>Cathode charge-transfer resistance</td><td>984.0 Ω</td><td>845.9 Ω</td><td>14.8 Ω</td><td>5.5 Ω</td><td>0.6 Ω</td><td>0.5 Ω</td></tr>
<tr><td>Mass transfer resistance</td><td>$97.4\ \Omega\ s^{-1/2}$</td><td>$13.6\ \Omega\ s^{-1/2}$</td><td>-</td><td>-</td><td>-</td><td>-</td></tr>
</table>

Supplementary Table 4 | Resistance components after fitting the EIS results to the equivalent circuits to EIS measurements of the full cells containing 3.3 mAh cm^{-2} LFP as cathode and 40 μm-thick pristine or naked Li metal foil as anode.

Supplementary References

[1] Liu, Y. *et al.* Lithium-coated polymeric matrix as a minimum volume-change and dendritefree lithium metal anode. *Nat. Commun.* **7**, 10992 (2016).

[2] Liang, Z. *et al.* Composite lithium metal anode by melt infusion of lithium into a 3D conducting scaffold with lithiophilic coating. *Proc. Natl. Acad. Sci. USA* **113**, 2862-2867 (2016).

[3] Lin, D. *et al.* Layered reduced graphene oxide with nanoscale interlayer gaps as a stable host for lithium metal anodes. *Nat. Nanotechnol.* **11**, 626-632 (2016).

[4] Zhang, Y. *et al.* High-capacity, low-tortuosity, and channel-guided lithium metal anode. *Proc. Natl. Acad. Sci. USA* **114**, 3584-3589 (2017).

[5] Hwang, J.-Y., Park, S.-J., Yoon, C.S. & Sun, Y.-K. Customizing a Li–metal battery that survives practical operating conditions for electric vehicle applications. *Energy Environ. Sci.* **12**, 2174-2184 (2019).

[6] Lin, D. *et al.* Three-dimensional stable lithium metal anode with nanoscale lithium islands embedded in ionically conductive solid matrix. *Proc. Natl. Acad. Sci. USA* **114**, 4613-4618 (2017).

[7] Lee, H., Song, J., Kim, Y.J., Park, J.K. & Kim, H.T. Structural modulation of lithium metalelectrolyte interface with three-dimensional metallic interlayer for high-performance lithium metal batteries. *Sci. Rep.* **6**, 30830 (2016).

[8] Song, J., Lee, H., Choo, M.J., Park, J.K. & Kim, H.T. Ionomer-liquid electrolyte hybrid ionic conductor for high cycling stability of lithium metal electrodes. *Sci. Rep.* **5**, 14458 (2015).

[9] Wang, H., Lin, D., Liu, Y., Li, Y. & Cui, Y. Ultrahigh-current density anodes with interconnected Li metal reservoir through overlithiation of mesoporous AlF_3 framework. *Sci. Adv.* **3**, e1701301 (2017).

[10] Maeyoshi, Y. *et al.* Long-term stable lithium metal anode in highly concentrated sulfolanebased electrolytes with ultrafine porous polyimide separator. *ACS Appl. Mater. Interfaces* **11**, 25833-25843 (2019).

[11] Chen, Y. *et al.* Steric effect tuned ion solvation enabling stable cycling of high-voltage lithium metal battery. *J. Am. Chem. Soc.* **143**, 18703-18713 (2021).

[12] Yu L*, et al.* A Localized high-concentration electrolyte with optimized solvents and lithium difluoro(oxalate)borate additive for stable lithium metal batteries. *ACS Energy Lett.* **3**, 20592067 (2018).

[13] Yu, Z. *et al.* Molecular design for electrolyte solvents enabling energy-dense and longcycling lithium metal batteries. *Nat. Energy* **5**, 526-533 (2020).

[14] Wang, H. *et al.* Dual-solvent Li-ion solvation enables high-performance Li-metal batteries. *Adv. Mater.* **33**, e2008619 (2021).

[15] Fan, X. *et al.* Non-flammable electrolyte enables Li-metal batteries with aggressive cathode chemistries. *Nat. Nanotechnol.* **13**, 715-722 (2018).

[16] Xue, W. *et al.* Ultra-high-voltage Ni-rich layered cathodes in practical Li metal batteries enabled by a sulfonamide-based electrolyte. *Nat. Energy* **6**, 495-505 (2021).

[17] Liu, L. *et al.* Free-standing hollow carbon fibers as high-capacity containers for stable lithium metal anodes. *Joule* **1**, 563-575 (2017).

[18] Wang, S.H. *et al.* Stable Li metal anodes via regulating lithium plating/stripping in vertically aligned microchannels. *Adv. Mater.* **29**, 1703729 (2017).

[19] Ren, X. *et al.* Enabling high-voltage lithium-metal batteries under practical conditions. *Joule* **3**, 1662-1676 (2019).

[20] Liu, Q. *et al.* Sustained-release nanocapsules enable long-lasting stabilization of Li anode for practical Li-metal batteries. *Nanomicro Lett.* **12**, 176 (2020).

[21] Chen, H. *et al.* Free-standing ultrathin lithium metal–graphene oxide host foils with controllable thickness for lithium batteries. *Nat. Energy* **6**, 790-798 (2021).

[22] Markevich, E., Salitra, G., Chesneau, F., Schmidt, M. & Aurbach, D. Very stable lithium metal stripping–plating at a high rate and high areal capacity in fluoroethylene carbonate-based organic electrolyte solution. *ACS Energy Lett.* **2**, 1321-1326 (2017).

[23] Suo, L. *et al.* Fluorine-donating electrolytes enable highly reversible 5-V-class Li metal batteries. *Proc. Natl. Acad. Sci. USA* **115**, 1156-1161 (2018).

[24] Wang, Z. *et al.* An anion-tuned solid electrolyte interphase with fast ion transfer kinetics for stable lithium anodes. *Adv. Energy Mater.* **10**, 1903843 (2020).

[25] Wang, W. *et al.* Stable cycling of high-voltage lithium-metal batteries enabled by highconcentration FEC-based electrolyte. *ACS Appl. Mater. Interfaces* **12**, 22901-22909 (2020).

[26] Yoo, D.J., Yang, S., Kim, K.J. & Choi, J.W. Fluorinated aromatic diluent for highperformance lithium metal batteries. *Angew. Chem. Int. Ed.* **59**, 14869-14876 (2020).

[27] Zhao, Q. *et al.* Upgrading carbonate electrolytes for ultra-stable practical lithium metal batteries. *Angew. Chem. Int. Ed.* e202116214 (2021).

[28] Zhang, W. *et al.* Engineering wavy-nanostructured anode interphases with fast ion transfer kinetics: toward practical Li-metal full batteries. *Adv. Funct. Mater.* **30**, 2003800 (2020).
